# Abstracts from the Virtual Symposium “Signal Transduction at the Blood–Brain Barriers”

**DOI:** 10.1186/s12987-021-00286-9

**Published:** 2021-12-17

## A1 SARS-CoV-2 spike protein induces brain pericyte immunoreactivity in absence of productive viral infection

### Natija Aldib^1,2,3^, Rayan Khaddaj-Mallat^1,2^, Anne-Sophie Paquette^1,3^, Aymeric Ferreir^2,3^, Sarah Lecordier^1,3^, Maxime Bernard^1,3^, Armen Saghatelyan^2,3^, Ayman ElAli^1,3^

#### ^1^Neuroscience Axis, Research Center of CHU de Quebec—Universite Laval, Quebec City, QC; Canada; ^2^Research Center CERVO, Quebec City, QC, Canada; ^3^Department of Psychiatry and Neuroscience, Faculty of Medicine, Universite Laval, Quebec City, QC, Canada

##### Correspondence: Ayman ElAli - ayman.el-ali@crchudequebec.ulaval.ca

###### *Fluids and Barriers of the CNS *2021, 18(1): A1

COVID-19 is a respiratory disease caused by severe acute respiratory syndrome coronavirus-2 (SARS-CoV-2). COVID-19 pathogenesis causes vascular-mediated neurological disorders via still elusive mechanisms. SARS-CoV-2 infects host cells by binding to angiotensin-converting enzyme 2 (ACE2) that recognizes the viral spike (S) protein. Brain pericytes express ACE2 at the neurovascular interface, outlining their possible implication in microvasculature injury in COVID-19. Yet, pericyte responses to SARS-CoV-2 is not clear. Here we report that ACE2 expression in human brain vascular pericytes is highly dynamic and is increased upon S protein stimulation. Pericytes exposed to S protein underwent phenotypic changes translated by increased expression of contractile and myofibrogenic proteins and an altered intracellular calcium dynamic. Furthermore, S protein induced lipid peroxidation, oxidative and nitrosative stress in pericytes as well as triggered an immune reaction, which was potentiated by hypoxia, a condition associated with vascular comorbidities. S protein exposure combined with hypoxia enhanced the production of pro-inflammatory cytokines. Finally, we found that S protein could reach the mouse brain via the intranasal route and that reactive ACE2-expressing pericytes are recruited to the damaged tissue undergoing fibrotic scarring in a mouse model of cerebral multifocal micro-occlusions. Our data demonstrate that the released S protein is sufficient to mediate pericyte immunoreactivity, which may contribute to microvasculature injury in absence of a productive viral infection.

**Grant support:** This work was supported by grants from the Neuroscience Thematic Research Center (CTRN) (#DR127915), the Natural Sciences and Engineering Research Council of Canada (NSERC) (#RGPIN-2017-06119), the Fonds de recherche du Quebec - Sante (FRQS) (#35170), and the Canadian Institutes of Health Research (CIHR) (#169062).

## A2 Investigating avenues for glioblastoma therapy with a microfluidic blood–brain barrier co-culture model

### Karin Danz, Lena Gammel, Saskia Altmaier, Julia Majer, Thorsten Knoll, Ina Meiser and Sylvia Wagner

#### Fraunhofer Institute for Biomedical Engineering IBMT, Sulzbach/Saar, Germany

##### Correspondence: Karin Danz- karin.danz@ibmt.fraunhofer.de.

##### *Fluids and Barriers of the CNS *2021, 18(1): A2

Glioblastomas are the most common primary brain tumours in adults with a median overall survival of only ~ 15 months. As in other oncological research, many approaches are being investigated and numerous new or improved treatment options are in development or undergoing clinical trials. However, the advances in glioblastoma treatment overall have been less pronounced than for other cancers. One major hurdle is that due to its localization, potential therapeutic agents have to be able to overcome the blood–brain barrier. To evaluate exosomes as a vehicle for targeted delivery in glioblastoma reduction, we are establishing a microfluidic model combining a hiPS-derived blood–brain barrier compartment with glioblastoma spheroids on the brain-side of the setup and a therapeutic organoid releasing targeted exosomes on the blood-side of the setup. We show that each cell-based component can be reliably cultivated in this triple-culture setup for up to 5 days, allowing the evaluation of therapeutic effects over an extended timeframe. The design of the system enables medium based sampling and downstream analysis during the experimental procedure as well as the removal and individual analysis of all cellular components after the experimental period. This provides the basis for a multi-facetted analysis of the exosome transport across the different fluidic and cellular compartments as well as allows detailed investigations on the effects of therapeutic exosomes on both the targeted glioblastoma structure and the biological barrier that has to be overcome in-between.

## A3 Cellular and subcellular spatiotemporal characterization of the neurovascular unit after ultrasound modulation

### Elisa Goncalves de Andrade^1^, Rikke Hahn Kofoed^2^, Kullervo Hynynen^2,3,4^, Isabelle Aubert^2,5,*^ and Marie-Eve Tremblay^1,6,7,8,9,*^

#### ^1^Division of Medical Sciences, University of Victoria, Victoria, Canada; ^2^Biological Sciences and Brain Sciences Research Program, Sunnybrook Research Institute, Toronto, Canada; ^3^Physical Sciences, Sunnybrook Research Institute, Toronto, Canada; ^4^Department of Medical Biophysics, Temerty Faculty of Medicine, University of Toronto, Canada; ^5^Department of Laboratory Medicine and Pathobiology, University of Toronto, Toronto, Canada; ^6^Axe Neurosciences, Centre de Recherche du CHU de Quebec, Universite de Laval, Quebec City, Canada; ^7^Neurology and Neurosurgery Department, McGill University, Montreal, Canada; ^8^Department of Molecular Medicine, Universite de Laval, Quebec City, Canada; ^9^Department of Biochemistry and Molecular Biology, University of British Columbia, Vancouver, Canada

*co-senior authors.

##### Correspondence: Marie-Eve Tremblay - evetremblay@uvic.ca

###### *Fluids and Barriers of the CNS* 2021, 18(1): A3

Transcranial magnetic resonance image-guided focused ultrasound combined with intravenous microbubbles (MRIgFUS) has emerged as a minimally invasive technology to transiently modulate blood–brain barrier (BBB) permeability, increasing transport and passive diffusion of therapeutic drugs from the blood to the brain. FUS, with and without microbubbles, has been associated with increased hippocampal neurogenesis and phagocytosis of amyloid-beta by glial cells in mouse models. Nevertheless, the effects of MRIgFUS on the NVU components remain largely unknown. Such critical data identifying outcomes on endothelial cells, basement membrane, pericytes, astrocytes, microglia, perivascular macrophages and neurons, is required to test the hypothesis that MRIgFUS transiently modulates the ultrastructure of BBB and NVU, crucial to inform overall brain homeostasis. We propose to provide insights into this structural modulation by conducting a cellular and subcellular spatiotemporal characterization of the BBB and NVU in the adult mouse hippocampus at 1, 24, and 96 h(s) post-MRIgFUS. These time points will be used to characterize the BBB and NVU state both during and after FUS induced BBB modulation. Using focused ion-beam scanning electron microscopy, we will investigate changes at the cellular (i.e. number of contacts, BBB coverage area) and subcellular (i.e. density of vacuoles, phagosomes, lysosomes, altered mitochondria, density vesicles and caveolae) levels of all NVU components. In the current presentation, we illustrate and discuss the ultrastructural analysis methodology, which will be used in this project. This investigation will allow us to establish the best criteria fit to characterize cellular and subcellular changes after MRIgFUS, crucial to inform future mechanistic studies.

**Grant Support:** This work was supported by the Canadian Institutes of Health Research (grants FRN-168906 to I.A. and FDN-154272 to K.H.), the National Institute of Biomedical Imaging and Bioengineering (RO1-EB003268, to K.H.), the Temerty Chair in Focused Ultrasound Research at Sunnybrook Health Sciences Centre, and Natural Sciences and Engineering Research Council (NSERC) Discovery grant (M-E. T.). Additional funding was received from the FDC Foundation, the WB Family Foundation, Gerald and Carla Connor, the Canada Research Chair Program (I.A. and M-E. T.). R.H.K. received a Postdoctoral Fellowship from the Alzheimer Society of Canada and a Carlsberg Internationalisation Fellowship (CF20-0379).

## A4 Novel hiPSC based spheroid model of the blood–brain barrier

### Sanjana Mathew^1^, Sabrina Oerter^2^, Winfried Neuhaus^3^, Andreas Brachner^3^, Jörg Piontek^4^, Marco Metzger^1,2^, Antje Appelt-Menzel^1,2^

#### ^1^University Hospital Wurzburg, Chair Tissue Engineering and Regenerative Medicine, Würzburg, Germany; ^2^Fraunhofer Institute for Silicate Research ISC, Translational Center Regenerative Therapies TLC-RT, Würzburg, Germany;^3^AIT Austrian Institute of Technology, Center Health and Bioresources, Vienna, Austria; ^4^Charite Berlin, Institute of Clinical Physiology, Berlin, Germany

##### Correspondence: Sanjana Mathew - sanjana.mathew@uni-wuerzburg.de

###### *Fluids and Barriers of the CNS* 2021, 18(1): A4

Various in vitro models of the blood–brain barrier (BBB) based on primary or immortalized human cells have been developed over the years [1] however, they do not show characteristic paracellular tightness as seen in vivo. The differentiation of human induced pluripotent stem cells (hiPSC) into brain capillary endothelial cells (BCECs) offers a promising approach to circumvent this [2–4]. Routinely used transwell based models lack direct neuro vascular cellular interactions, leading to focus in the development of advanced models. Advances in tissue engineering have shown that a 3D BBB spheroid model can be generated spontaneously using immortalized or primary cells [5–7]. We previously established protocols to generate a 2D co-culture BBB model using the transwell system [8]. To improve the drawbacks of this model, we developed an advanced BBB spheroid model using hiPSC-derived BCECs, primary astrocytes and primary pericytes. Characterization of our model via freeze fracture electron microscopy showed the presence of tight junctions. Additionally, upregulation in Junction adhesion molecule-1, ATP–binding cassette transporter ABCA-1, Vascular endothelial growth factor-A, Claudin-11 and Platelet endothelial cell adhesion molecule-1 compared to 2D transwells was observed at mRNA level. We were also able to confirm the presence and localization of Zonula-Occludes-1, Transferrin receptor, Glucose transporter 1 and P-Glycoprotein via immunofluorescence. Our advanced model mimics in vivo physiology with direct cellular contact and offers the potential for the development of personalized therapeutics.

**Grant Support:** This work was supported by Horizon 2020 Project Im2pact (Proposal number 807015–2) and stipends from the Graduate school of life sciences, University Würzburg.

**References**

1. Appelt-Menzel A, Oerter S, Mathew S, Haferkamp U, Hartmann C, Jung M et al. Human iPSC-Derived Blood–Brain Barrier Models: Valuable Tools for Preclinical Drug Discovery and Development? Curr Protoc Stem Cell Biol. 2020;55(1):e122. https://doi.org/10.1002/cpsc.122.
2. Lippmann ES, Azarin SM, Kay JE, Nessler RA, Wilson HK, Al-Ahmad A et al. Derivation of blood–brain barrier endothelial cells from human pluripotent stem cells. Nat Biotechnol. 2012;30(8):783–91. https://doi.org/10.1038/nbt.2247.
3. Lippmann ES, Al-Ahmad A, Azarin SM, Palecek SP, Shusta EV. A retinoic acid-enhanced, multicellular human blood–brain barrier model derived from stem cell sources. Sci Rep. 2014;4:4160. https://doi.org/10.1038/srep04160.
4. Qian T, Maguire SE, Canfield SG, Bao X, Olson WR, Shusta EV et al. Directed differentiation of human pluripotent stem cells to blood–brain barrier endothelial cells. Sci Adv. 2017;3(11):e1701679. https://doi.org/10.1126/sciadv.1701679.
5. Eilenberger C, Rothbauer M, Selinger F, Gerhartl A, Jordan C, Harasek M et al. A Microfluidic Multisize Spheroid Array for Multiparametric Screening of Anticancer Drugs and Blood–Brain Barrier Transport Properties. Adv Sci (Weinh). 2021;8(11):e2004856. https://doi.org/10.1002/advs.202004856.
6. Cho CF, Wolfe JM, Fadzen CM, Calligaris D, Hornburg K, Chiocca EA et al. Blood–brain-barrier spheroids as an in vitro screening platform for brain-penetrating agents. Nat Commun. 2017;8:15,623. https://doi.org/10.1038/ncomms15623.
7. Urich E, Patsch C, Aigner S, Graf M, Iacone R, Freskgard PO. Multicellular self-assembled spheroidal model of the blood brain barrier. Sci Rep. 2013;3:1500. https://doi.org/10.1038/srep01500.
8. Appelt-Menzel A, Cubukova A, Gunther K, Edenhofer F, Piontek J, Krause G et al. Establishment of a Human Blood–Brain Barrier Co-culture Model Mimicking the Neurovascular Unit Using Induced Pluri- and Multipotent Stem Cells. Stem Cell Reports. 2017;8(4):894–906. https://doi.org/10.1016/j.stemcr.2017.02.021.

## A5 Human in vitro models of the neurodevelopmental rare disease SYNGAP1 Syndrome indicate a blood–brain barrier phenotype

### Anna Gerhartl^1^, Andreas Brachner^1^, Lena Czeloth^1^, Heinz-Peter Friedl^1^, Olaf Ries^2^, Jenny Kintzel^3^, Nerea Llamosas^4^, Matthias Jung^3^, Gavin Rumbaugh^4^, Winfried Neuhaus^1^

#### ^1^AIT—Austrian Institute of Technology GmbH, Center Health and Bioresources, Competence Unit Molecular Diagnostics, Vienna, Austria; ^2^Institute of Medical Genetics and Applied Genomics, University of Tubingen, Tubingen, Germany; ^3^Institute of Biochemical Chemistry, Medical Faculty of the Martin Luther University Halle-Wittenberg, Halle (Saale), Germany; ^4^Department of Neuroscience, Scripps Research, Jupiter, Florida, USA

##### Correspondence: Winfried Neuhaus – winfried.neuhaus@ait.ac.at

###### *Fluids and Barriers of the CNS* 2021, 18(1): A5

SYNGAP1 is a high-risk genetic locus for neurodevelopmental disorders (NDD). It is estimated that about 1% of intellectual disability (ID) cases might be caused by severe de novo variants in SYNGAP1 resulting in haploinsufficiency, which leads to a defined phenotype characterized by ID with epilepsy [termed Mental Retardation-Type 5(MRD5); OMIM#603384]. Currently, about 400 cases of SYNGAP1 syndrome have been diagnosed worldwide. In addition, the published data underlined the notion that pathogenic SYNGAP1 loss-of-function variants are both highly penetrant and sufficient to cause NDDs [1]. The focus of research so far has been very much centered on neuronal changes and damage. Since the blood–brain barrier (BBB) can play a crucial role both for drug delivery and for communication during the course of the disease between the periphery and the CNS, as well as within the CNS, the first step was to investigate the influence of SYNGAP1 in human in vitro models of the BBB. Comparison of BBB in vitro models differentiated from human induced pluripotent stem cells (wild-type, SYNGAP1-knock out, missense mutation Leu323Arg) revealed significant differences in the paracellular barrier, supported at the molecular level by DNA methylation data, RNAseq, high throughput qPCR (including a comprehensive analysis of tight junction proteins, ABC- and SLC transporters, receptors, etc.), western blotting and immunofluorescence microscopy, allowing conclusions on the underlying mechanisms. In addition, the models were applied for transport studies with statins, which have been proposed as treatment options in case studies. In conclusion, the data suggest an intrinsic alteration of BBB function in SYNGAP1 disorder.

**Grant Support:** This work was supported by Leon & friends e.V. (Leons BBB) to W.N.

**References**

1. Kosmicki JA, Samocha KE, Howrigan DP, Sanders SJ, Slowikowski K, Lek M et al. Refining the role of de novo protein-truncating variants in neurodevelopmental disorders by using population reference samples. Nat Genet. 2017;49(4):504–10. https://doi.org/10.1038/ng.3789.

## A6 Elucidation of a possible digoxin-binding site in the substrate-binding pocket of P- glycoprotein 

### Lasse Saaby^1,2^ and Birger Brodin^2^

#### ^1^Bioneer A/S, Denmark; ^2^Department of Pharmacy, University of Copenhagen, Denmark

##### Correspondence: Lasse Saaby - lsa@bioneer.dk

###### *Fluids and Barriers of the CNS 2021*, 18(1): A6

P-glycoprotein (P-gp, ABCB1) is an efflux transporter expressed at the major barriers of the human body e.g. the small intestine, blood–brain barrier, liver, kidney, placenta and testis. P-gp utilizes ATP hydrolysis to transport a wide range of structurally diverse substrates across cell plasma membranes against their concentration gradient. In this way, P-gp affects the absorption, distribution and excretion of substrate drug compounds. At present, the crystal structure of human P-gp has not been resolved and therefore, the molecular mechanisms underlying the function of P-gp and how substrates interact with the transporter is not fully understood. At least two different transport-enabling drug-binding sites have been described; the H- and the R-sites, named after the observed interacting substrates Hoechst 33,342 and Rhodamine 123, respectively [1, 2]. Based on in vitro experimentation on murine and human P-gp combined with data from in silico pharmacophore modeling, the existence of a third distinct binding site for the well-known P-gp substrate digoxin has been hypothesized [3, 4]. In this talk, our efforts to explore the possible existence of a digoxin-binding site different from the putative H- and R-binding sites previously described for P-gp will be presented. This will cover the use of a polarized confluent cell monolayer model over-expressing human P-gp to study interactions between digoxin and known P-gp substrates and inhibitors.

**Grant Support:** This work was supported by the Danish Agency for Higher Education and Science (LS).

**References**

1. Shapiro AB, Ling V. Positively cooperative sites for drug transport by P-glycoprotein with distinct drug specificities. Eur J Biochem. 1997;250(1):130–7. https://doi.org/10.1111/j.1432-1033.1997.00130.x.
2. Martin C, Berridge G, Higgins CF, Mistry P, Charlton P, Callaghan R. Communication between multiple drug binding sites on P-glycoprotein. Mol Pharmacol. 2000;58(3):624–32. https://doi.org/10.1124/mol.58.3.624.
3. Ledwitch KV, Barnes RW, Roberts AG. Unravelling the complex drug-drug interactions of the cardiovascular drugs, verapamil and digoxin, with P-glycoprotein. Biosci Rep. 2016;36(2). https://doi.org/10.1042/BSR20150317.
4. Bocci G, Moreau A, Vayer P, Denizot C, Fardel O, Parmentier Y. New insights in the in vitro characterisation and molecular modelling of the P-glycoprotein inhibitory promiscuity. Eur J Pharm Sci. 2018;121:85–94. https://doi.org/10.1016/j.ejps.2018.04.039.

## A7 Laser processing of lab-on-chip microfluidic platforms for BBB modeling

### Cristina Elena Staicu^1,2^, Florin Jipa^2^, Anca Bonciu^3^, Emanuel Axente^2^, Beatrice Mihaela Radu^1^, Felix Sima^2^

#### ^1^Department of Anatomy, Animal Physiology and Biophysics, Faculty of Biology, University of Bucharest, 91–95 Splaiul Independenței, 050095 Bucharest, Romania; ^2^Center for Advanced Laser Technologies (CETAL), National Institute for Laser, Plasma and Radiation Physics, 409 Atomiștilor St., 77125 Măgurele, Romania; ^3^Department of Lasers, National Institute for Laser, Plasma and Radiation Physics, 409 Atomiștilor St., 77125 Măgurele, Romania

##### Correspondence: Cristina Elena Staicu - elena.staicu@inflpr.ro

###### *Fluids and Barriers of the CNS* 2021, 18(1): A7

The blood–brain barrier (BBB) is the main structure involved in the transport of substances between the peripheral blood and the cerebral parenchyma. With the attempt to avoid animal sacrifice and physiological differences, it became critical to develop BBB models able to recreate in vivo physiology and pathological conditions, in particular for pharmacokinetic testing applications. Lab-on-a-chip technology has been extensively used in recent years to mimic BBB in microfluidic chips. Due to their complex structures, materials used for manufacturing of microfluidic devices require special attention in terms of both biocompatibility and functionality. Photosensitive glasses, fluoropolymers and polydimethylsiloxane are herein investigated for BBB mimicking as they exhibit good biocompatibility and appropriate physico-chemical and optical characteristics, suitable for molecular analyses of junction proteins. Laser direct writing, thermal treatments and subsequent chemical wet etching were applied to fabricate true 3D hollow channels inside glass or molding glass systems for replication of polymers. Endothelial bEnd.3 cells were then grown in dedicated chips with complex configurations adapted for BBB modeling. MTS viability and proliferation tests, scanning electron and immunofluorescence microscopy revealed good material biocompatibility and adequate 3D microenvironments for cell culturing.

**Grant Support:** This work was supported by the Romanian Ministry of Education and Research, CCCDI-UEFISCDI, project numbers PN-III-P4-ID-PCE-2020-1952 (PCE8/2021), PN-III-P2-2.1-PED-2019-4657.

## A8 Using human iPSC-derived brain endothelial-like cells to evaluate the influence of quiescence-related protein expression changes on blood–brain barrier functions

### Lindsey M. Williams^1^, Takashi Fujimoto^2^, Riley Weaver^1^, William A. Banks^1,3^, and Michelle A. Erickson^1,3^

#### ^1^Veterans Affairs Puget Sound Health Care System, Geriatrics Research Education and Clinical Center, Seattle, WA, USA; ^2^Nagasaki University Graduate School of Biomedical Sciences, Department of Neurosurgery, Nagasaki, Nagasaki, Japan; ^3^University of Washington School of Medicine, Department of Medicine, Division of Gerontology and Geriatric Medicine, Seattle, WA, USA

##### Correspondence: Lindsey Williams - linds.williams@outlook.com.

###### *Fluids and Barriers of the CNS* 2021, 18(1): A8

The brain endothelial cells (BEC) comprising the blood–brain barrier (BBB) possess both barrier and interface functions to selectively control molecular transfer between the blood and brain. Although these functions are difficult to fully recapitulate in vitro, human iPSC-derived models of BECs (iBECs) acquire many key specialized features of BECs, such as high trans-endothelial electrical resistance (TEER), expression of tight junction proteins (TJPs), and functional nutrient, protein, and drug transport systems. We have discovered that iBECs become less proliferative over time spent in culture, enabling evaluation of changes to BBB properties as BECs adopt a more quiescent phenotype. Firstly, we find that iBECs exhibit changes in TJP expression that are not correlated with changes in TEER. While TEER can be higher or lower in more quiescent iBECs as compared to their more proliferative counterparts, we find that, consistently, claudin-5 expression is decreased, zona occludens-1 expression is increased, and occludin expression remains unchanged. Further, we find that iBECs exhibit changes in expression of glucose metabolism/transport-related enzymes that reflect the adoption of endothelial metabolic quiescence. Specifically, downregulation of glycolysis is indicated by reduced 6- phosphofructo-2 kinase/fructose-2,6-biphosphatase-3 (PFKFB3), hexokinase-2 (HK-2), and monocarboxylatetransporter-1 (MCT-1), while glucose transporter-1 (GLUT-1), the predominant glucose transporter at the BBB, is upregulated. Interestingly, despite increased GLUT-1 expression in iBECs’ more quiescent state, bidirectional transendothelial glucose transport kinetics are significantly decreased. Our findings support that iBECs are well-suited for investigations into the relationships between BEC proliferation and BBB functions to provide mechanistic insight into the pathology of neurological diseases such as Alzheimer’s disease.

**Grant support:** This work was supported by the NIH (R21 ES029657 AG065928) and the US Department of Veteran’s Affairs.

## A9 The Effectiveness of Antagonists of Four Histamine Receptor Types and Inhibitor of RhoA in Reversing in Histamine-Induced Transient Blood–Brain Barrier Opening

### Uğur Akcan^1^, Arzu Temizyürek^2^, Fidan Şeker Polat^3^, Canan Uğur Yılmaz^4^, Gözde Demirci^5^, İrem Akbulak^1^, Ayşe Selin Üzbe^2^, Pakize Pelin Üzbe^2^, Funda Yağcı Acar^5^, Mehmet Kaya^6^, Bülent Ahıshalı^7^

#### ^1^Department of Neuroscience, School of Medicine, Koc University, Istanbul, Turkey; ^2^School of Medicine, Koc University, Istanbul, Turkey; ^3^Department of Obstetrics and Gynecology, Feinberg School of Medicine, Northwestern University, Chicago, USA; ^4^Department of Pharmaceutical Biosciences, Uppsala University, Uppsala; ^5^Department of Chemistry, School of Science, Koc University, Istanbul; ^6^Department of Physiology, School of Medicine, Koc Univesity, Istanbul; ^7^Department of Histology and Embryology, School of Medicine, Koc University, Istanbul

##### Correspondence: Uğur Akcan - uakcan17@ku.edu.tr

###### *Fluids and Barriers of the CNS* 2021, 18(1): A9

Blood–brain barrier (BBB) which creates a major obstacle for transport of molecules into the brain can be opened by various treatments for effective drug delivery into the brain parenchyma, however its subsequent closure is also crucial to limit the alterations that can potentially occur in neuronal microenvironment by substances. In this study, we tested the efficacy of blocking histamine receptors (HR) and RhoA in reversing histamine-induced BBB opening. Following intravenous histamine injection (10 mg/kg), mice were treated with antagonists, hydroxyzine (H1R, 3 mg/kg), cimetidine (H2R, 10 mg/kg), ciproxifan (H3R, 3 mg/kg) and JNJ-7777124 (H4R, 20 mg/kg), and Rho A inhibitor fasudil (10 mg/kg). Blood–brain barrier permeability to molecules was evaluated by fluorescent detection of intravenously administered albumin-Alexa Fluor-594 (1%) and cadaverine Alexa-Flour-488 (1%), respectively in brain parenchyma. The alterations in HR expression were detected by RT PCR. Group differences were determined by one-way ANOVA followed by Tukey test. Differences between the means were considered significant if p < 0.05. Treatment with hydroxyzine significantly decreased fluorescence intensity of albumin-Alexa Fluor-594 in brain following histamine induced BBB opening (p < 0.01), while there were no significant changes in immunofluorescence intensity of cadaverine Alexa-Flour-488 by HR antagonists and fasudil. No significant changes were noted in HR expression following administration of HR antagonists and fasudil to histamine-treated mice. Our results suggest that treatment with H1R antagonist hydroxyzine restores histamine-induced BBB disruption which emphasizes its potential use as a novel means of closing transiently opened BBB to protect the neuronal microenvironment against potentially toxic blood-borne substances in brain drug delivery applications.

**Grant Support:** This work was supported by TUBİTAK.

## A10 Design and development of ultrasound responsive nanocarriers as neuroblastoma nanotherapeutics

### Arathyram Ramachandra Kurup Sasikala, Hanene Ali-Boucetta

#### Nanomedicine, Drug Delivery & Nanotoxicology Lab, The School of Pharmacy, College of Medical and Dental Sciences, University of Birmingham, Edgbaston, Birmingham, B15 2TT, UK

##### Correspondence: Arathyram Ramachandra Kurup Sasikala -a.ramachandrakurupsasikala@bham.ac.uk, Hanene Ali-Boucetta - h.aliboucetta@bham.ac.uk.

###### *Fluids and Barriers of the CNS* 2021, 18(1): A10

Non-invasive cancer treatment approaches using light [1], magnetic field [2], electric field [3], and ultrasound [4] combined with drug delivery systems have gained an increased attention in recent times. This is due to the potential improvement in treatment efficiency as well as reduced recovery time, minimal infection, and scar formation. Neuroblastoma is the most common extracranial solid tumour in childhood, but the available treatments are challenged by high rates of resistance, recurrence, and progression of the tumour [5]. Here we report the development of ultrasound (US) sensitive carboxyl functionalised piezoelectric barium titanate nanoparticles conjugated with an anticancer drug doxorubicin (DOX) in a pH-dependent manner. These unique nanoparticles transduce ultrasound signals to electrical signals to permeate the cell membrane through nano-electroporation [6] allowing the effective penetration of therapeutics and enhancing drug accumulation inside the cancer cells. In addition, they exhibited Second Harmonic Generation (SHG) imaging property to noninvasively monitor the uptake of the nanoparticles. Thus, the theranostics effects of these nanosystems were evaluated in the SHSY-5Y human neuroblastoma cells. SHG imaging showed that the pulsed US improved the penetration of these nanocarriers and the delivery of DOX in both 2D monolayers and 3D tumour spheroids cultures resulting in an enhanced anticancer effect. As a future perspective, these uniquely modified piezoelectric nanoparticles can be exploited noninvasively to electrically permeate the cell membranes of the BBB and enable therapeutic molecules to enter the brain.

**Grant Support:** This research has been funded by the European Union’s Horizon 2020 Research and Innovation Programme under the Marie Sklodowska-Curie Grant Agreement No 898170.

**References**

1. Sasikala ARK, Unnithan AR, Thomas RG, Batgerel T, Jeong YY, Park CH et al. Hexa-functional tumour-seeking nano voyagers and annihilators for synergistic cancer theranostic applications. Nanoscale. 2018;10(41):19568–78. https://doi.org/10.1039/c8nr06116e.
2. Sasikala ARK, Unnithan AR, Thomas RG, Ko SW, Jeong YY, Park CH, Kim CS. Multifaceted implantable Anticancer device for potential postsurgical breast cancer treatment: a single platform for synergistic inhibition of local regional breast cancer recurrence, surveillance, and healthy breast reconstruction. Advanced Functional Mat. 2018;28 (8), 1704793
3. Mun EJ, Babiker HM, Weinberg U, Kirson ED, Von Hoff DD. Tumor-Treating Fields: A Fourth Modality in Cancer Treatment. Clin Cancer Res. 2018;24(2):266–75. https://doi.org/10.1158/1078-0432.CCR-17-1117.
4. Grainger SJ, Serna JV, Sunny S, Zhou Y, Deng CX, El-Sayed ME. Pulsed ultrasound enhances nanoparticle penetration into breast cancer spheroids. Mol Pharm. 2010;7(6):2006–19. https://doi.org/10.1021/mp100280b.
5. Gurunathan S, Jeyaraj M, Kang MH, Kim JH. Anticancer Properties of Platinum Nanoparticles and Retinoic Acid: Combination Therapy for the Treatment of Human Neuroblastoma Cancer. Int J Mol Sci. 2020;21(18). https://doi.org/10.3390/ijms21186792.
6. Sharabi S, Bresler Y, Ravid O, Shemesh C, Atrakchi D, Schnaider-Beeri M et al. Transient blood–brain barrier disruption is induced by low pulsed electrical fields in vitro: an analysis of permeability and trans-endothelial electric resistivity. Drug Deliv. 2019;26(1):459–69. https://doi.org/10.1080/10717544.2019.1571123.

## A11 Quantifying brain penetration of antibodies using vascular depletion and multiplexed MRM mass spectrometry

### Christie E. Delaney, Alexandra T. Star, Wen Ding, Danica B. Stanimirovic and Arsalan S. Haqqani

#### Human Health Therapeutics Research Centre, National Research Council, Ottawa, Canada

##### Correspondence: Christie E. Delaney - Christie.Delaney@nrc-cnrc.gc.ca

###### *Fluids and Barriers of the CNS* 2021, 18(1): A11

Many methods for the quantification of brain penetration use levels of therapeutic antibodies in CSF or total brain as an indication of brain parenchyma exposure. To quantify the actual penetration of blood–brain barrier (BBB)-crossing antibodies into the brain parenchyma we developed a filtration method to separate the vessels from the brain parenchyma of rats systemically injected with the BBB-crossing antibody FC5hFc. To validate the method, multiple reaction monitoring (MRM) mass spectrometry was used to quantify the amount of FC5hFc in the vessel enriched and vessel-depleted brain fractions. MRM is a highly sensitive method that provides absolute quantification and sequence-specific identification that can be multiplexed to measure multiple antibodies and biomarkers in the same reaction. In the rat brain, FC5hFc was predominantly trafficked out of the vasculature and into the brain parenchyma. A non-BBB-crossing control antibody, A20.1hFc, was found to be sequestered to the vessel-enriched fraction, with less than 0.1% of the antibody detectable in the parenchyma. Selected biomarkers of the brain parenchyma and vasculature were monitored in the multiplexed MRM analysis to validate that the vessel markers were found in very low levels in the brain parenchyma, demonstrating that the vessels were depleted from the parenchyma with a high level of purity. This method was adapted for a study of non-human primates to quantify brain penetration of therapeutic antibodies, and to demonstrate the effect on biomarkers predictive of drug response, using multiplexed MRM.

## A12 The role of calpain inhibitor-I in NF-κB pathway in an in vitro blood–brain barrier model under inflammatory conditions

### İrem Akbulak^1,2^, Deniz Altunsu^1,2^, Ecem Ayvaz^1,2^, Arzu Temizyurek^2^, Bulent Ahıshalı^3^, Mehmet Kaya^2,4^

#### ^1^Koc University, Graduate School of Health Sciences, Istanbul, Turkey; ^2^Koc University Research Center for Translational Medicine, Istanbul, Turkey; ^3^Koc University, School of Medicine, Department of Histology and Embryology, Istanbul, Turkey; ^4^Koc University, School of Medicine, Department of Physiology, Istanbul, Turkey

##### Correspondence: İrem Akbulak - iakbulak20@ku.edu.tr

###### *Fluids and Barriers of the CNS* 2021, 18(1): A12

The barrier-type endothelial cells in the brain play a critical role in maintaining central nervous system homeostasis by regulating the movement of substances between blood and brain parenchyma. Nuclear factor-kappa B (NF-κB), a transcription factor which regulates inflammatory signaling pathways when activated by lipopolysaccharide (LPS), is known to participate in endothelial dysfunction in various diseases associated with blood–brain barrier (BBB) disruption. In this study, we aimed to investigate whether inhibiting the proteolytic degradation of the inhibitor of κB (IκB) by using calpain inhibitor-I could alleviate LPS induced BBB disruption in an in vitro BBB model established by direct-contact endothelial (bEnd.3)/astrocyte co-culture. Inflammation was induced by LPS administration (100 ng/mL) to bEnd.3 cells on the luminal side of Transwell inserts during a 24-h incubation. Afterwards, cells were treated with calpain inhibitor-I (5, 10, 20, 50 μM) for 4 h. The integrity and permeability of BBB were assessed by measurements of transepithelial/transendothelial electrical resistance (TEER) and sodium fluorescein (NaFl) leakage, respectively. TEER values were higher in LPS plus 20 μM calpain inhibitor-I group compared to both LPS and LPS plus 50 μM calpain inhibitor-I groups (p < 0.05). Treatment with 20 μM calpain inhibitor-I following LPS exposure significantly reduced NaFl leakage into the lower chamber of Transwell compared to LPS (p < 0.05), LPS plus 5 μM calpain inhibitor-I and LPS plus 10 μM calpain inhibitor-I treatment groups (p < 0.01). Our results suggest that calpain inhibitor-I is a promising agent for alleviation of inflammation-related BBB disruption, therefore may account for a novel therapeutic approach in inflammation and sepsis.

## A13 Immunocytochemical and functional characteristics of cultured mouse brain endothelial cells in inflammatory conditions

### Beata Barabasi^1^, Lilla Barna^1^, Fruzsina Walter^1^, Ana Raquel Santa Maria^1^, Andras Harazin^1^, Erzsebet Melinda Toth^2^, Miklos Santha^2^, Zsofia Hoyk^1^, Maria A. Deli^1^

#### ^1^Institute of Biophysics and ^2^Institute of Biochemistry, Biological Research Centre, Szeged, Hungary

##### Correspondence: Zsofia Hoyk – hoyk.zsofia@brc.hu, Maria A. Deli - deli.maria@brc.hu

Hypertriglyceridemia is linked to atherosclerosis, increased production of pro-inflammatory cytokines including tumor necrosis factor-α (TNFα) and interleukin-6 (IL6), and may alter the function of the blood–brain barrier (BBB). Brain endothelial cells were isolated from transgenic mice which overexpress the human APOB-100 gene and show chronic hypertriglyceridemia. The cells were analyzed under control conditions and following pro- and anti-inflammatory cytokine treatments mimicking different inflammatory signals. Our experiments focused on the following functional and immunohistochemical characteristics of the BBB: para- and transcellular permeability, transendothelial electric resistence (TEER), fluorescence intensity of tight junction protein (claudin-5, occludin, ZO-1) and P-glycoprotein (Pgp) immunolabeling. Cultured brain endothelial cells from APOB-100 transgenic mice showed an increased paracellular permeability compared to wild types under control conditions, which was further increased after IL-6, IL-6 + IL-10 and TNFα + IL-1β treatment. TEER was lower in transgenic endothelial cells than in wild-type cells without cytokine treatment. This difference was enhanced following IL-6, IL-10 and IL-6 + IL-10 administration. In accordance with these observations an increased whole cell occludin and ZO-1 and a decreased Pgp immunofluorescence intensity was detected in transgenic cells compared to wild types in control conditions. Following different cytokine treatments the observed changes were partially enhanced. Pgp function assay showed no significant difference in control cells, but all cytokine application resulted in a decrease in Pgp activity in the transgenic group. Our results suggest that inflammatory conditions linked to hypertriglyceridemia may damage BBB function by altering Pgp activity and the expression of some crucial proteins in brain capillary endothelial cells.

**Grant support:** This work was supported by GINOP-2.3.2-15-2016-00060.

## A14 Role of the angiogenic factor Angiopoietin-2 in myeloid cell recruitment into the inflamed CNS

### Kristina Berve^1,2^, Giuseppe Locatelli^1^, Britta Engelhardt^1^

#### ^1^Theodor-Kocher-Institute, University of Bern, Switzerland; ^2^Marie Sklodowska-Curie Innovative Training Network ENTRAIN

##### Correspondence: Kristina Berve—Kristina.berve@tki.unibe.ch

###### *Fluids and Barriers of the CNS* 2021, 18(1): A14

In multiple sclerosis (MS) resident and infiltrating myeloid cells are the predominant inflammatory cells within lesions of the CNS. Studies have shown a detrimental role of infiltrating monocytes aggravating disease progression and outcome in a mouse model of MS. To study the mechanisms involved in migration of myeloid cells into the CNS we here focus on the growth factor Angiopoietin-2 (Ang-2). Ang-2 is part of the Ang/Tie system critical for the regulation of vascular development, maturation and stability during embryogenesis as well as for vessel remodelling during pathology. We established a transgenic mouse model with inducible endothelial cell specific overexpression of Ang-2 (EC-Ang2). These animals slowly develop a chronic disease characterized by progressive blood vessel enlargement, increased vascular leakage and accumulation of myeloid cells in numerous peripheral tissues. We crossbred EC-Ang2 mice with the myeloid cell knock-in reporter mice CX3CR1 + /GFP/CCR2 + /RFP generating EC-Ang2/CX3CR1 + /GFP**/**CCR + /RFP mice allowing to distinguish CNS-resident GFP + and inflammatory RFP + myeloid subsets. Quantitative analysis of immune cell infiltrates using flow cytometry revealed a strong increase of CCR2 + infiltrating macrophages as well as lymphocytes in the brain and spinal cord of EC-Ang2 mice during steady state. Interestingly, male EC-Ang-2 mice displayed a higher number of infiltrating immune cells in comparison to age-matched female transgenic mice. Analysis of precise cell localization in brain sections by immunofluorescence microscopy suggests that Ang-2 recruits myeloid cells mostly to the CNS interfaces, since CCR2 + and CCR2/CX3CR1 + double positive cells accumulated mainly in the leptomeninges and within blood vessels. The CNS parenchyma on the other hand was devoid of those cells. Subjecting female EC-Ang2 mice to active EAE, a common mouse model for MS, revealed however that moderate Ang-2 overexpression does not lead to significantly increased CNS immune cell infiltrates and consequently does not aggravate EAE disease course. Nevertheless, the role of Ang-2 in neuroinflammation could be masked by the strong inflammatory environment created by our adjuvant-based EAE model and furthermore dependent on sex and Ang-2 concentration gradually increasing over time. In summary, endothelial Ang-2 overexpression results in immune cell accumulation in the CNS during steady state, while its role during neuroinflammation remains to be investigated.

**Grant support:** Marie Sklodowska-Curie Innovative Training Network ENTRAIN.

## A15 Treatment with mesenchymal stem cells (MSCs) decreases microgliosis and microglial CCL2 release in EAE mice

### Antonio d’Amati^1,4^, Mariella Errede^1^, Tiziana Annese^1^, Valentina Petrosino^2^, Giovanna Longo^1^, Francesco Girolamo^1^, Ignazio de Trizio^1,3^, Antonio Uccelli^2,5^, Nicole Kerlero de Rosbo^2^, Daniela Virgintino^1^

#### ^1^Department of Basic Medical Sciences, Neuroscience, and Sensory Organs, University of Bari School of Medicine, Bari, Italy; ^2^Department of Neurosciences, Ophthalmology, Genetics, Maternal and Child Health, University of Genoa, Genoa, Italy; ^3^Intensive Care Unit, Department of Intensive Care, Regional Hospital of Lugano, Ente Ospedaliero Cantonale, Lugano, Switzerland; ^4^Department of Emergency and Organ Transplantation, Pathology Section, University of Bari School of Medicine, Bari, Italy; ^5^IRCCS Ospedale Policlinico San Martino, Genoa, Italy

##### Correspondence: Antonio d’Amati - antonio.damati@uniba.it

###### *Fluids and Barriers of the CNS* 2021, 18(1): A15

The cerebral neocortex of experimental autoimmune encephalomyelitis (EAE)-affected mice reveals multiple areas of demyelination associated with blood–brain barrier (BBB) disruption and inflammation. The administration of mesenchymal stem cells (MSCs), as demonstrated in previous studies, significantly reduces astrogliosis and neuroinflammation in EAE. In this study we investigated the cellular sources of CCL2, a chemokine involved in monocyte recruitment and in mechanisms of BBB breakdown, with the aim of further examining the pathogenesis of neocortex EAE and the possible remedial effects of MSC treatment in this condition. Macrophage/microglia markers and microglia-specific markers, i.e. TMEM119 and SALL1 were investigated, combined with CCL2, by immunohistochemistry (IHC) and dual RNAscope IHC/in situ hybridization. The results showed that the primary source of CCL2 release in EAE is represented by hypertrophic microglia, which crowd around neocortex neurons and get in contact with BBB-impaired microvessels. EAE-affected mice treated with MSC administration, revealed decreased CCL2 expression, restored BBB structure and reduced microgliosis. The observations of our study further explored the pathogenetic mechanisms involved in neocortex BBB disruption and neuroinflammation, and demonstrated beneficial effects after MSC treatment, suggesting possible therapeutic implications in neuroinflammatory and neurodegenerative diseases.

**Grant Support:** This work was supported by Italian Multiple Sclerosis Foundation (FISM) and by Apulia Foundation.

## A16 Collagen VI/NG2 vascular relations in human developing brain and glioblastoma

### De Trizio Ignazio^1,2^, Annese Tiziana^1^, D’Amati Antonio^1,3^, Girolamo Francesco^1^, Errede Mariella^1^, Virgintino Daniela^1^

#### ^1^Department of Basic Medical Sciences, Neuroscience and Sensory Organs, Human Anatomy and Histology Unit, University of Bari School of Medicine, Bari, Italy; ^2^Department of Internal Medicine, Regional Hospital of Lugano, Ente Ospedaliero Cantonale, Lugano, Switzerland; ^3^Department of Emergency and Organ Transplantation, Pathology Section, University of Bari School of Medicine, Bari, Italy

##### Correspondence: Ignazio de Trizio - ignazio.detrizio@gmail.com

###### *Fluids and Barriers of the CNS* 2021, 18(1): A16

Despite different therapeutic strategies, human glioblastoma (GBM) remains the brain neoplasia with the worst prognosis. It is among the most vascularized tumors and many efforts have been made to target glioblastoma neovascularization. In recent years growing evidence is available about the underestimated role of extracellular matrix proteins in the tumor microenvironment. Collagen type VI (COLVI) is an uncommon form of collagen with an increased expression in many pathological conditions, including the GBM, where it prevails as a vascular basal lamina (VBL) component. Among the numerous cell receptors for COLVI, neuron/glia antigen 2 (NG2) gains interest due to its overexpression in glioblastoma, both in tumor cells and in tumoral vessel pericytes. The putative role of the COLVI/NG2 axis in normal brain vascularization, along with its links to glioma vessel neo-formation, remains an open field of investigation. In this study, we have analyzed COLVI/NG2 immunolocalization and relations associated with vascular cell proliferation and spreading in the human developing brain and glioblastoma samples, by Real Time-PCR and immunofluorescence confocal analysis. The results show that COLVI and NG2, which are barely detectable in the normal adult brain, characterize the growing brain microvessels, appearing regularly and tightly intercalated on the vessel wall. In glioblastoma, a remarkable presence of COLVI, that forms a multilayered VBL to glomeruloid vessels, parallels the overwhelming contribution of NG2-expressing pericytes to the vessel wall. The COLVI/NG2 association identifies distinctive perivascular niches, where pericytes proliferate and possibly give origin to pericyte sprouting and vascular tube formation, thus contributing to the tumoral vascular network. These observations shed new light on the possible role of the COLVI/NG2 interplay in glioblastoma vessel proliferation to further identify diagnostic and therapeutic tools.

## A17 Methamphetamine induces oxidative and endoplasmic reticulum stress in blood–brain barrier pericytes

### Nikolai Fattakhov, Silvia Torices, Minseon Park, and Michal Toborek

#### Department of Molecular Biology and Biochemistry, Miller School of Medicine, University of Miami, Miami, USA

##### Correspondence: Nikolai Fattakhov - nfattakhov@med.miami.edu

###### *Fluids and Barriers of the CNS* 2021, 18(1): A17

Methamphetamine (METH) is a psychostimulant that causes neurologic and psychiatric abnormalities. There is no effective therapeutic intervention developed targeting cerebrovascular toxicity of METH. Recent studies have suggested that oxidative stress induced by METH may play a vital role in neuroinflammation and increased blood–brain barrier (BBB) permeability but the mechanisms remain poorly understood. The current study focused on analyzing the changes in endoplasmic reticulum (ER) stress markers and oxidative damage in METH-treated human brain vascular pericytes. Exposure of human brain vascular pericytes to METH stimulated the formation of mitochondrial reactive oxygen species (ROS), as detected by quantitative analyses of MitoSOX Red fluorescence signal intensity*.* Considering that altered redox balance has a major impact on ER folding capacity, we further examined whether METH alters protein expression levels of ER stress/unfolded protein response (UPR) markers. Western blot analysis showed increased expression of molecular sensor ATF6 in human brain pericytes treated with METH for 24 h, suggesting the involvement of ATF6 pathway in pericyte damage caused by METH. Our study suggests that METH may establish a vicious cycle as excess generation of ROS that leads to an increase in protein misfolding. These results contribute to the understanding of the molecular mechanisms of METH-induced damage to human brain pericytes and the role of cellular stress in this process.

**Grant Support:** Supported by the National Institutes of Health (NIH), grants DA044579, DA039576, DA040537, DA050528, DA047157, MH128022, MH122235, MH072567, and HL126559. Dr S. Torices was supported by an American Heart Association (AHA) postdoctoral fellowship (20POST35211181).

## A18 Increased expression of chemokine receptor CXCR4 in astrocyte endfeet at the neurovascular unit from mesial temporal lobe epilepsy patients with hippocampal sclerosis

### Elif Fidan^1^, Isabela Kiesewetter Zandavalli^1^, Felix Bader^2^, Karl H. Plate^1^, Patrick Harter^1^, Stefan Günther^3^, Thomas Freiman^2^, Jürgen Konczalla^2^, Kavi Devraj^1^, Stefan Liebner^1^

#### ^1^Goethe University Clinic, Institute of Neurology (Edinger Institute), Frankfurt am Main, Germany; ^2^Goethe University Clinic, Department of Neurosurgery, Frankfurt am Main, Germany; ^3^Max Planck Institute for Heart and Lung Research, Bioinformatics and Deep Sequencing Platform, Bad Nauheim, Germany

##### Correspondence: Elif Fidan - elif.fidan@kgu.de

###### *Fluids and Barriers of the CNS* 2021, 18(1): A8

Mesial temporal lobe epilepsy (MTLE) is the most common form of refractory epilepsy, characterized by spontaneous recurrent seizures. Impairment of the blood–brain barrier (BBB) has been attributed to the formation and/or progression of the disease, but a detailed knowledge of the molecular changes at the BBB and the neurovascular unit (NVU) is missing. To characterize BBB properties affected by epilepsy or affecting the progress of epilepsy, human microvessel fragments, resembling the NVU, of morphologically inconspicuous cortex and epileptic hippocampus of MTLE patients were compared. RNA-Seq revealed significantly regulated genes in epileptic hippocampus, being related with cadherin signaling, negative regulation of canonical Wnt signaling, vascular permeability, and inflammatory response. Among the latter, CXCR4 was found to be overexpressed in epileptic and sclerotic hippocampus. Its overexpression has been validated by real-time polymerase chain reaction (qRT-PCR). Immunostaining revealed increased CXCR4 expression specifically in the sclerotic hippocampus that localizes to astrocyte endfeet at the NVU. Our results demonstrate the pathological change in the astrocyte endfeet at the NVU in sclerosis. To mechanistically examine the role of astrocytic CXCR4 expression for BBB function astrocytes overexpressing CXCR4 will be co-cultured with mouse brain microvascular endothelial cells (MBMECs) and transendothelial electrical resistance (TEER) measurements will be performed. Preliminary TEER experiments revealed that treatment of astrocytes with CXCR4 antagonist AMD3100 resulted in improved barrier properties of MBMECs, suggesting its protective role on the BBB.

**Grant Support:** This project was supported by LOEWE CePTER.

## A19 Effects of breast cancer chemotherapy on the blood–brain barrier in vitro

### Rebecca Gebert^1^, Carolin J. Curtaz^2^, Patrick Meybohm^1^, Achim Wöckel^2^, Malgorzata Burek^1^

#### ^1^University of Würzburg, Department of Anaesthesiology, Intensive Care, Emergency and Pain Medicine, Würzburg, Germany; ^2^University of Würzburg, Department of Gynecology and Obstetrics, Würzburg, Germany

##### Correspondence: Rebecca Gebert - Gebert_R@ukw.de

###### *Fluids and Barriers of the CNS* 2021, 18(1): A19

**Background:** The molecular subtype of breast cancer influences the pattern of its metastatic progression. Human epidermal growth factor receptor-2 overexpressing (HER2+) breast cancer is associated with an increased risk of brain metastasis. First line chemotherapy consists of anti-HER2-monoclonal antibodies such as trastuzumab and pertuzumab, which have limited penetration into the CNS. After administration of HER2 antibodies, the risk of developing brain metastases is increased, even if the primary disease and visceral metastases respond to the therapy. This raises the question of whether treatment with HER2 antibodies increases the permeability of the blood–brain barrier (BBB) and enables circulating tumor to enter the brain more easily. In case of brain metastases, small-molecule tyrosine kinase inhibitors against HER2, such as tucatinib, promise a more effective treatment due to their better BBB penetration. In the present study, we want to examine the effects of different chemotherapy treatments for breast cancer on the BBB.

**Methods:** We used an in vitro human BBB model that we had validated in previous studies as a co-culture of brain-like endothelial cells, BLECs (CD34+ cells-derived) with brain pericytes. We measured the paracellular permeability for fluorescein and performed the immunofluorescence staining, MTT Assay, Western blot and mRNA analysis.

**Results:** In our in vitro model, chemotherapy did not significantly change the paracellular permeability. While no changes occurred on protein level, the qPCR showed an insignificant tendency towards reduced mRNA levels of the tight junction proteins claudin-5 and occludin. Tight junctions in immunostaining with anti-claudin-5 antibodies appeared to be more diffuse after chemotherapy. Treatment with tucatinib significantly increased the metabolic activity of the BLECs in the MTT assay.

**Conclusion:** The first experimental in vitro results show several effects of breast cancer therapy on BBB properties. However, in vivo data are required to demonstrate the clinical relevance of our results.

## A20 Protocadherin-Gamma-C3 in Glioblastoma multiforme—Effects on surface markers in brain microvascular endothelial cells

### Mara Lauer^1^, Patrick Meybohm^1^, Carsten Hagemann^2^, Malgorzata Burek^1^

#### ^1^University of Würzburg, Department of Anaesthesiology, Intensive Care, Emergency and Pain Medicine, Würzburg, Germany; ^2^University of Würzburg, Department of Neurosurgery, Tumour Biology Laboratory, Würzburg, Germany

##### Correspondence: Mara Lauer - Lauer_M2@ukw.de

###### *Fluids and Barriers of the CNS* 2021, 18(1): A20

The blood–brain barrier (BBB), consisting of microvascular endothelial cells surrounded by pericytes and astrocytes, regulates metabolite transfer to the central nervous system. Glioblastoma multiforme (GBM) is a highly aggressive and invasive primary brain tumor with a median survival time of around 15–20 months. It can alter the permeability of the BBB, causing dysfunction and deregulation of the BBB. Protocadherin-Gamma-C3 (PCDHGC3), a transmembrane glycoprotein of cadherin superfamily that mediates calcium-dependent cell adhesion, is overexpressed in GBM and in brain microvascular endothelial cells (BMECs). As we recently showed, a knockout of PCDHGC3 in BMECs leads to an altered BBB function. Here we generated a PCDHGC3-knockout in the GBM cell line U343. We used the human BMECs hCMEC/D3 in co-culture with knockout and wild type GBM cells and analyzed the gene expression in BMECs using the TaqMan Array Human Cell Surface Markers. The expression of CSF1R, CD63, CD83, VCAM1 and VWF was decreased in hCMEC/D3 the coculture with GBM cells compared to hCMEC/D3 monoculture, while the expression of CD1D, CD38, CD69, CD96 and KLRD1 was increased due to the co-culture independently of the PCDHGC3 expression status. The co-culture with the PCDHGC3 knockout GBM cell line led selectively to an increased expression of S100A8, TNFRSF4, TNFRSF8, UBC and a decreased expression of KLRD1. A better understanding of these changes and the effects of PCDHGC3 on the BBB and GBM can help to develop better treatment options that can lead to higher survival rates in GBM patients.

## A21 Impact of traumatic brain injury on blood–brain barrier properties: role of neuropeptide Y

### Ricardo A. Leitão^1,2,3^, José Luis Alves^2,3,4^, Ana Luisa Bernardo^1,2,3^, Carlos A. Fontes-Ribeiro^1,2,3^, Ana Paula Silva^1,2,3^

#### ^1^Institute of Pharmacology and Experimental Therapeutics, Faculty of Medicine, University of Coimbra, Portugal; ^2^Coimbra Institute for Clinical and Biomedical Research (iCBR), Faculty of Medicine, University of Coimbra, Portugal; ^3^Center for Innovative Biomedicine and Biotechnology (CIBB), University of Coimbra, Portugal; ^4^Neurosurgery Service, Coimbra Hospital and University Centre (CHUC), Portugal

##### Correspondence: Ricardo Leitão - ricardoaleitao@gmail.com

###### *Fluids and Barriers of the CNS* 2021, 18(1): A21

Traumatic brain injury (TBI) is a devastating condition that affects millions of people worldwide. Its most described features are excitotoxicity, brain edema and blood–brain barrier (BBB) disruption, highlighting the involvement of neurovascular unit in TBI outcomes. Some authors have demonstrated that neuropeptide Y (NPY) can have neuroprotective effects in some neuropathological conditions, mainly through inhibition of excitotoxicity. Yet, its role on TBI has never been investigated. Therefore, the aim of this work was to uncover the role of NPY on TBI-induced neurovascular alterations. To address our goal, we used a closed-head weight drop TBI model (male Sprague–Dawley rats, 3-month-old). NPY (100 μg/animal) was administrated intranasally 30 min after injury, and animals were euthanized 48 h or 7 days after the TBI. We observed a clear increase in albumin expression in the hippocampus at both time-points as well as a downregulation of claudin-5, indicating BBB disruption. Moreover, upregulation of adhesion molecules (ICAM-1) was observed in both ipsilateral and contralateral, but an increase in CD4 protein levels (marker of T-lymphocytes) was only identified in the contralateral side. Further, we observed a dual effect on vessel coverage by astrocytes, with an increase in macrovessels and less coverage in microvessels in both ipsilateral and contralateral hippocampi. Interestingly, the administration of NPY was able to counteract all the outcomes observed after TBI. We concluded that BBB are highly affected by TBI, which could potentiate the transmigration of peripheral immune cells. Additionally, we uncover NPY as a promising tool to control the negative effects induced by TBI.

**Grant Support:** This work was supported by Project Pest-C/SAU/UI3282/2013-2014, CNC.IBILI UID/NEU/04539/2019, UIDB/04539/2020 and UIDP/04539/2020 (CIBB) with national funds PT2020/COMPETE 2020 and POCI (FCOMP-01-0124-FEDER-028417, POCI-01-0145-FEDER-007440, Centro 2020 Regional Operational Programme: BRAINHEALTH 2020 (CENTRO-01-0145-FEDER-000008).

## A22 Autoimmunity to brain blood vessels in patients with encephalitis: antibody binding pattern and functional implications

### Lucie Y Li^1^, Malgorzata Burek^2^, Jakob Kreye^3,4^, S Momsen Reincke^3,4^, Cesar Cordero-Gomez,^3,4^, Elisa Sanchez-Sendin^3,4^, Patrick Meybohm^2^, Paula Charlotte Barthel^1^, Harald Pruss^3,4^, Markus Holtje^1^

#### ^1^Institute of Integrative Neuroanatomy Berlin, Charite-Universitätsmedizin Berlin, corporate member of Freie Universität Berlin, Humboldt-Universität zu Berlin, and Berlin Institute of Health, Berlin, Germany; ^2^Department of Anaesthesiology, Intensive Care, Emergency and Pain Medicine, University Hospital Würzburg; Würzburg, Germany; ^3^Department of Neurology and Experimental Neurology, Charite-Universitätsmedizin Berlin, corporate member of Freie Universität Berlin, Humboldt-Universität Berlin, and Berlin Institute of Health, Berlin, Germany; ^4^German Center for Neurodegenerative Diseases (DZNE) Berlin, Berlin, Germany

##### Correspondence: Lucie Y Li - lucie-yuanting.li@charite.de

###### *Fluids and Barriers of the CNS* 2021, 18(1): A22

Autoantibodies against neuronal receptors and synaptic proteins play a key role in the pathogenesis of autoimmune encephalitis, which can lead to certain neuropsychiatric disorders. In order to reach their targets in the central nervous system and to exert their pathological effects on neural circuits, they have to overcome the blood–brain barrier (BBB). Antibodies directed against blood vessels play a putative role in this mechanism. The antibody repertoire of patients with autoimmune encephalitis was screened for antibodies directed against blood vessels. The identification of target antigens and functional analysis on the in vitro BBB model aimed to demonstrate how these antibodies might contribute to the pathogenesis.

Human recombinant monoclonal autoantibodies from patients diagnosed with autoimmune encephalitis were tested for binding to brain blood vessels using indirect immunofluorescence. In a repertoire of 159 monoclonal autoantibodies from 5 patients, we were able to detect 7 antibodies with a pronounced binding to brain blood vessels. The immunohistochemical characterization revealed specific patterns, such as preferential binding to certain vessel calibers or additional reactivity to non-vessel neuronal targets. Antigen identification as well as initial tests on the BBB model have already provided evidence for alterations in permeability.

In summary, antibodies from autoimmune encephalitis patients show differential binding patterns to brain blood vessels. Initial tests on the in vitro BBB model indicate antibody effects on the transendothelial electrical resistance and permeability. Further systematic testing and antigen identification should further elucidate the role of antibodies directed against blood vessels in the pathogenesis.

## A23 Regulation of blood–brain barrier integrity by microbiome-associated dietary methylamines

### Lesley Hoyles^1^, Matthew G. Pontifex^2^, Ildefonso Rodriguez-Ramiro^2,3^, M. Areeb Anis-Alavi^4^, Tom Snelling^5^, Egle Solito^6,7^, Sonia Fonseca^8^, Ana L. Carvalho^8^, Simon R. Carding^2,8^, Michael Müller^2^, Robert C. Glen^5,9^, David Vauzour^2^ and Simon McArthur^4^

#### ^1^Department of Biosciences, School of Science and Technology, Nottingham Trent University, Clifton, Nottingham, UK; ^2^Norwich Medical School, University of East Anglia, Norwich, UK; ^3^Metabolic Syndrome Group, Madrid Institute for Advanced Studies (IMDEA) in Food, Madrid, E28049, Spain; ^4^Institute of Dentistry, Barts & the London School of Medicine & Dentistry, Blizard Institute, Queen Mary University of London, London, UK; ^5^Faculty of Medicine, Department of Metabolism, Digestion and Reproduction, Imperial College London, London, UK; ^6^William Harvey Research Institute, Barts & the London School of Medicine & Dentistry, Queen Mary, University of London, London, UK; ^7^Dipartimento di Medicina molecolare e Biotecnologie mediche, Federico II University, Naples, Italy; ^8^The Gut Microbes and Health Research Programme, The Quadram Institute, Norwich Research Park, Norwich, UK; ^9^Centre for Molecular Informatics, Department of Chemistry, University of Cambridge, Cambridge, UK

##### Correspondence: Simon McArthur - s.mcarthur@qmul.ac.uk

###### *Fluids and Barriers of the CNS* 2021, 18(1): A23

Communication between the gut microbiota and the brain is primarily mediated via soluble microbe-derived metabolites, but the details of this pathway remain poorly defined. Methylamines produced by microbial metabolism of dietary choline and L-carnitine have received attention due to their proposed association with vascular disease, but their effects upon the cerebrovascular circulation have not hitherto been studied. We have investigated how physiologically relevant concentrations of the dietary methylamine trimethylamine (TMA) and its host-derived oxidation product trimethylamine *N*-oxide (TMAO) affect blood–brain barrier (BBB) integrity.

In vitro studies revealed that while TMA dose-dependently impaired BBB function, TMAO exhibited a biphasic response, physiologically relevant concentrations reducing and supraphysiological doses enhancing permeability. Microarray analysis indicated that TMA activated pathways characteristic of cellular stress responses, while TMAO upregulated a number of pathways associated with cytoskeletal rearrangement and actin bundle formation. Notably, TMAO upregulated expression of the major tight junction regulator annexin A1. Further analysis of this pathway using hCMEC/D3 cells stably expressing shRNA sequences targeting annexin A1 showed this protein to be a major mediator of TMAO actions. Acute treatment of mice with TMAO enhanced BBB integrity within 2 h, and was able to restore the BBB permeability defect induced by LPS administration. Chronic treatment of mice for two months with TMAO reduced signs of BBB integrity damage caused by long-term sub-acute LPS treatment and prevented object recognition memory impairment. Physiologically-relevant concentrations of the dietary methylamine TMAO can beneficially modulate BBB integrity, emphasising the BBB as an interface in the gut microbiota-brain axis.

**Grant support:** This work was supported by Alzheimer’s Research UK Pilot Grant No. ARUKPPG2016B-6696 and MRC grant MR/L01632X.

## A24 Dickkopf-1 impairs structural and functional recovery after ischemic stroke

### Romain Menet^1,2^, Sarah Lecordier^1,2^, Maxime Bernard^1,2^ and Ayman ElAli^1,2^

#### ^1^Department of Psychiatry and Neuroscience, Faculty of Medicine, Universite Laval; ^2^Research Center of CHU de Quebec-Universite Laval, Quebec City, QC, Canada

##### Correspondence: Ayman ElAli - Ayman.El-Ali@crchudequebec.ulaval.ca

###### *Fluids and Barriers of the CNS* 2021, 18(1): A24

Stroke constitutes a major cause of death and disability in the industrialized world. Stroke, which occurs as a result of a sudden obstruction within a cerebral artery due to an embolus or thrombus, accounts for the majority of cases. We have previously shown that upon stroke, the canonical Wnt pathway, which plays key roles in regulating neurovascular functions, is deregulated. Interestingly, Dickkopf-1 (DKK1), a secreted inhibitor of the pathway, has been shown to be induced after stroke in patients and animal models. Although DKK1 levels have been shown to increase, its implication in stroke pathobiology and therapy remains unknown. Our study aims to elucidate the role of DKK1 following ischemic stroke. For this purpose, TOPGAL reporter mice and inducible DKK1 mice will be used to dissect DKK1 role in regulating canonical Wnt pathway throughout injury and repair upon stroke. Furthermore, DKK1 will be neutralized in the sub-acute phase using a pharmacological approach to explore the therapeutic potential its inhibition of neurovascular repair. Our results indicate that ectopic DKK1 induction aggravates infarct and oedema sizes as well as well as impaired motor functions after stroke. Furthermore, DKK1 induction induced neuronal degeneration, while preventing neurogenesis, neuronal maturation and angiogenesis. These changes were accompanied by increased levels of glutamate in the blood circulation. Importantly, pharmacological neutralization of DKK1 using WAY262611 improved structural and functional recovery after stroke. These findings indicate that DKK1 plays a key role in stroke pathobiology and its neutralization constitutes a clinically relevant approach to enhance neurovascular repair after stroke.

**Grant Support:** This work was supported by grants from the Natural Sciences and Engineering Research Council of Canada (NSERC) (Grant # RGPIN-2017-06119), the *Fonds de recherche du Québec—Santé* (FRQS), the Heart and Stroke Foundation of Canada (Grant # G-18-0022118), and the Canadian Institutes of Health Research (CIHR) (Grant # 426287).

## A25 Implications of Blood–Brain Barrier leakage in Alzheimer’s disease

### Geetika Nehra^1^, Ruedeemars Yubolphan^1,2^, Brittany Sulkowski^1^, Rachel Cooke^1^, Samantha Mullins^1^, Pornpun Vivithanaporn^2^, Bjoern Bauer^3^, Anika M. S. Hartz^1,4^

#### ^1^Sanders-Brown Center on Aging, University of Kentucky, USA; ^2^Department of Pharmacology, Mahidol University, Thailand; ^3^Department of Pharmaceutical Sciences, College of Pharmacy, University of Kentucky, USA; ^4^Department of Pharmacology and Nutritional Sciences, College of Medicine, University of Kentucky, USA

##### Correspondence: Anika Hartz - anika.hartz@uky.edu; Geetika Nehra - geetika.nehra@uky.edu

###### *Fluids and Barriers of the CNS* 2021, 18(1): A25

Blood–brain barrier leakage in Alzheimer’s disease (AD) contributes to AD pathology and cognitive decline but the onset and extent of blood–brain barrier leakage in AD remain to be defined. In this study, we evaluated these metrics in the 5xFAD mouse model of AD. Briefly, we examined 3-month-old, 6-month-old, and 12-month-old wild-type and 5xFAD mice for spatial memory impairment onset using Y-Maze and Morris Water Maze tests. Next, cranial windows were installed in 8-month-old, 12-month-old, and 16-month-old mice to assess barrier leakage in vivo via two-photon microscopy. Fluorescently labeled dextrans (3 kDa, 70 kDa) were injected and Z-stacks were acquired over 60 min following dextran injection. At the end of each imaging session, mice were euthanized and isolated hemispheres were immerse-fixed, sectioned and immunostained for collagen IV, a basement-membrane marker, to evaluate the extent of dextran leakage ex vivo via confocal microscopy. Our behavioral tests show that spatial memory is significantly impaired in 12-month-old 5xFAD mice compared to age-matched wild-type mice but no impairments were observe in 3-month-old or 6-month-old mice. We also observed instances of dextran leakage in 8-month-old 5xFAD mice but not in age-matched wild-type mice. Dextran leakage instances increased at 12 months and 16 months of age. In conclusion, our findings indicate that barrier leakage initiates prior to spatial memory impairment in 5xFAD mice and increases with aging. We are now identifying mechanisms that lead to barrier dysfunction in 5xFAD mice and if these mechanisms are also altered in postmortem human brain tissue sections.

**Grant support:** This work was supported by the NIH Grant 2R01AG039621 entitled: “Restoring Blood–Brain Function to Improve Cognition in Alzheimer's Disease (AD)”.

## A26 Methamphetamine induces cognitive decline via affecting the blood–brain barrier and aberrant proliferation and differentiation of neural progenitor cells

### Minseon Park^1^, Arkadiusz Liśkiewicz^2^, Andrzej Małecki^2^, and Michal Toborek^1,2^

#### ^1^Department of Biochemistry and Molecular Biology, University of Miami Miller School of Medicine, Miami, FL 33136; ^2^Institute of Physiotherapy and Health Sciences, The Jerzy Kukuczka Academy of Physical Education, Katowice 40-065, Poland

##### Correspondence: Minseon Park - MSPark@med.miami.edu, Michal Toborek - mtoborek@med.miami.edu, Andrzej Małecki - a.malecki@awf.katowice.pl

###### *Fluids and Barriers of the CNS* 2021, 18(1): A26

Methamphetamine (METH) abusers are prone to developing a variety of comorbidities, including cognitive disabilities, and the immunological responses have been recognized as an important component involved in the toxicity of this drug. In addition, maintaining an intact pool of neural progenitor cells (NPCs) is crucial for generating new and functionally active neurons. Our research has focused on the mechanisms of METH-induced cognitive decline via affecting differentiation and proliferation of NPCs. Exposure of mice to METH markedly increased the protein level of IL-1β in the hippocampus and induced a decline in spatial learning and memory. Importantly, METH-induced impaired spatial learning abilities were attenuated by coadministration of mouse IL-1 Trap, a dimeric fusion protein that incorporates the extracellular domains of both of the IL-1 receptor components required for IL-1 signaling. Furthermore, chronic exposure to METH resulted in aberrant differentiation and enhanced proliferation of NPCs. This latter effect was long-lasting as it was preserved ex vivo in NPCs isolated from the exposed mice over several passages in the absence of additional treatments. Further studies revealed that upregulation of the CXCL12-CXCR4 axis, leading to activation of downstream pAkt and pErk and phosphorylation of FOXO3 can be responsible for this effect. Our findings suggest a potential new therapeutic pathway for treatment of altered cognitive abilities that occur in METH abusing individuals.

**Grant support:** The study was supported in part by the National Science Centre (NSC) grant 2019/35/B/NZ7/03155 and the National Institutes of Health (NIH), grants DA050528, DA044579, DA039576, and DA040537.

## A27 Altered Protein Expression of ABC and SLC Transporters at the BBB and Brain Cortex of Familial Alzheimer’s Disease Animal Models

### Elena Puris^1^, Seppo Auriola^2^, Liudmila Saveleva^3^, Robin Hartman^4^, Izaque de Sousa Maciel^3^, Mikko Gynther^1^, Katja M. Kanninen^3^, Elizabeth C. M. de Lange^4^, Gert Fricker^1^

#### ^1^Ruprecht-Karls-University, Institute of Pharmacy and Molecular Biotechnology, Heidelberg, Germany; ^2^University of Eastern Finland, School of Pharmacy, Kuopio, Finland; ^3^University of Eastern Finland, A.I. Virtanen Institute for Molecular Sciences, Kuopio, Finland; ^4^Leiden University, Leiden Academic Centre for Drug Research, Division of Systems Biomedicine and Pharmacology, Predictive Pharmacology Group, Leiden, the Netherlands

##### Correspondence: Elena Puris - elena.puris@uni-heidelberg.de

In Alzheimer’s disease (AD), the function and expression of several membrane transporters mediating passage of solutes, i.e. nutrients and drugs, across the blood–brain barrier (BBB) and brain parenchymal cells have been reported to be altered. There is limited information on quantitative changes in the transporter protein expression in AD brains. Moreover, AD-related sex-specific alterations in transporter expression in the brain have not been studied. Animal models of AD have not been previously characterized in terms of the changes in transporter protein expression. Here, we studied the changes in absolute protein expression of five ATP binding cassette (ABC) and fourteen solute carrier (SLC) transporters at the BBB and brain cortical tissue in two commonly used animal models of AD such as TgF344-AD rats and 5xFAD mice as compared to age-matched wild-type controls. In addition, we investigated sex specific alterations in transporter expression in the brain cortical tissue of the TgF344-AD rats and 5xFAD mice. The liquid chromatography tandem mass spectrometry (LC–MS/MS)-based quantitative targeted absolute proteomic analysis revealed significant changes in protein expression of several transporters in the isolated brain microvessels and brain cortical tissue, which were model- and sex-specific. The protein expression in the investigated AD models was compared to published data in AD patients in order to evaluate the relevance of the models to mimic AD-related changes. The study brings new knowledge for the elucidation of molecular mechanisms underlying AD and provides a first insight into the optimal use of the animal models in AD drug development and drug delivery research.

**Grant support:** This work was supported by the Alexander von Humboldt Foundation, the Alzheimer Forschung Initiative e.V. (AFI), the Biocenter Finland and Biocenter Kuopio supporting the analytical chemistry laboratory.

## A28 Serum from breast cancer patients with primary cancer, visceral, bone or brain metastases and its effects on the blood–brain barrier in vitro

### Constanze Schmitt^1^, Carolin J. Curtaz^2^, Achim Wockel^2^, Patrick Meybohm^1^, Malgorzata Burek^1^

#### ^1^University of Würzburg, Department of Anaesthesiology, Intensive Care, Emergency and Pain Medicine, Würzburg, Germany; ^2^University of Würzburg, Department of Gynecology and Obstetrics, Würzburg, Germany

##### Correspondence: Constanze Schmitt - constanze.schmitt@stud-mail.uni-wuerzburg.de

###### *Fluids and Barriers of the CNS* 2021, 18(1): A28

Breast cancer often metastasizes to the lungs, bones, and brain. Brain metastases correlate with the worst overall survival, despite advances in diagnosis and therapy. The key event for the metastatic progression of breast cancer in the brain is the migration of cancer cells across the blood–brain barrier (BBB). The circulating cancer-derived factors can play a role in this process. We therefore collected serum samples from healthy donors, breast cancer patients with primary tumors, and breast cancer patients with brain, bone or visceral metastases and analyzed its effects on human in vitro BBB model derived from CD34 + cells in co-culture with pericytes. We performed the measurements of paracellular permeability, immunofluorescence staining, Western blot and real-time PCR on serum-treated cells. The increased paracellular permeability, accompanied by changes in the immunostaining of tight junction protein claudin-5, was observed in cells treated with sera from breast cancer patients with cerebral and bone metastases. The mRNA of claudin-5 and occludin was decreased, while the mRNA of efflux pumps was increased after treatment with sera from breast cancer patients with primary cancer, cerebral and visceral metastases. This was partially confirmed at the protein level. Our results show that sera from breast cancer cells with primary breast cancer or breast cancer metastases change the BBB properties differently. Changes in the BBB permeability and the transporter level in brain microvascular endothelial cells can lead to long-term consequences in the course of the disease. However, more in vitro and in vivo experiments are needed to identify the serum derived factors behind the observed effects.

## A29 Transcriptomics Profiling of Cerebral Amyloid Angiopathy Reveal Alterations in Tight Junctions and Inflammatory Genes

### Muyu Situ^1^ and Anuska V. Andjelkovic^1,2^

#### ^1^Neuroscience Graduate Program, University of Michigan, Ann Arbor, Michigan; ^2^Department of Pathology and Neurosurgery, University of Michigan Medical School, Ann Arbor, MI, USA

##### Correspondence: Muyu Situ - muyusitu@umich.edu

###### *Fluids and Barriers of the CNS* 2021, 18(1): A29

Cerebral Amyloid Angiopathy (CAA) pathology is defined by vascular hyperpermeability, inflammation, and lobar hemorrhages in the brain due to Blood Brain Barrier (BBB) dysfunction. However, genetic disparities underlying BBB dysfunction in CAA pathogenesis are not as well-studied and still relatively unknown. The goal of the study was to determine Aβ deposition in CAA contributes to alteration in TJ and pro-inflammatory gene expression consequently contributes to brain endothelial barrier dysfunction. Using mRNA-sequencing, we analyzed the transcriptome of microvascular vessels isolated from a murine model of CAA (Tg-SwDuI; Thy1 APPSwDutIowa) compared to aged match control mice. Old TgSwDuI mice (14–16 months) had 849 upregulated differentially expressed genes (DEGs) and 280 downregulated DEG) relative to their aged-matched control. While Old TgSwDuI mice had only 534 upregulated DEGs and 64 downregulated relative to Young TgSwDuI mice. Both RNA-sequencing data and gene set enrichment analysis pinpointed TJ-associated gene (*TJ1*, *PECAM-1*, etc.) and pro-inflammatory gene (*CSF3R, NFKBIA, SERPINE1,* etc.) alterations in old TgSwDuI mice compared to aged match control and Young TgSwDuI mice (5–6 months). Overall, we demonstrated novel genetic variations that facilitate BBB dysfunction in CAA disease pathology and may have significant implications in other vascular related aging and cerebrovascular disease.

**Grant Support:** This work was supported by NIH-R01 F048216.

## A30 S1 subunit of SARS-CoV-2 spike protein and Methamphetamine dysregulate human brain endothelial cells

### Michael Stangis^1^, Daniel Adesse^2^, and Michal Toborek^1^

#### ^1^Department of Biochemistry and Molecular Biology, University of Miami Miller School of Medicine, Miami, FL, 33136, USA; ^2^Laboratory of Structural Biology, Instituto Oswaldo Cruz, Fiocruz, Rio de Janeiro, RJ, CEP 21045–900, Brazil

##### Correspondence: Michael Stangis - mstangis@miami.edu

###### *Fluids and Barriers of the CNS* 2021, 18(1): A30

With the incredible variety of symptoms reported in individuals infected with COVID-19, including stroke and encephalopathy, the question of how the virus was able to influence the brain arose. Our work has shown that following infection with SARS-CoV-2, human brain endothelial cells show decreases in tight junction proteins. These effects are also seen in the short term (1–12 h) following exposure to just the S1 subunit of the SARS-CoV-2 Spike protein, with recovery and eventual over expression occurring following long-term exposure (48–72 h). This down regulation in tight junction proteins points toward a loss of blood brain barrier (BBB) integrity and would allow for the virus to more easily pass through the BBB and infect cells of the neurovascular unit. These changes in tight junction proteins appear to be further amplified when the human brain endothelial cells are co-exposed to Methamphetamine. Our data indicates additional dysregulation of mitochondrial respiration following exposure and co-exposure to the S1 subunit and/or methamphetamine in similar short-term timeframes to when tight junction proteins were downregulated, indicating a potential association. Current studies are also aiming to identify if inflammatory cytokines are produced within the same conditions and how these events may interact with one another to compromise BBB integrity.

**Grant Support:** This work was supported by the National Institutes of Health (NIH), grants DA044579, DA039576, DA040537, DA050528, DA047157, MH122235, MH072567, and HL126559. Dr. Adesse was supported by Fundacao Oswaldo Cruz (INOVA Fiocruz, grant number 48401984391807) and Fundacao de Amparo a Pesquisa do Estado do Rio de Janeiro (FAPERJ, SEI-260003/002704/2020).

## A31 Circadian clock disruption enhances endothelial toxicity of polychlorinated biphenyls

### Timea Teglas, Madison Taylor, Michal Toborek

#### Department of Molecular Biology and Biochemistry, Miller School of Medicine, University of Miami, Miami, USA

##### Correspondence: Timea Teglas - txt575@miami.edu

###### *Fluids and Barriers of the CNS* 2021, 18(1): A31

Polychlorinated biphenyls (PCBs) are stable, lipophilic, and highly toxic industrial products. Diet, including animal products, represents approximately 90% of PCB exposure. The chemical properties of PCBs ensure their resistance to degradation and accumulation in human and animal tissues. The metabolic effects of coplanar and non-coplanar PCBs are complex and impact the main organ systems and signaling pathways. In most species, including humans, the endogenous circadian clocks coordinate physiological, molecular, and behavioral 24-h rhythms. At the molecular level, circadian oscillators are based on an interlocked system of transcriptional-translational feed-back loops. Central to the molecular clock are a pair of transcription factors, namely, brain and muscle ARNT-like 1 (BMAL1) and circadian locomotor output cycles kaput (CLOCK). Both environmental (e.g., sleep disruption) and genetic factors (e.g., mutations or polymorphisms in circadian rhythm genes) can disrupt the circadian rhythms and influence pathogenesis and progression of a variety of diseases. In this work, we used *Bmal1* siRNA gene silencing for the modeling of circadian rhythm disruption, followed by exposure of endothelial cells to coplanar and non-coplanar PCBs. Our results indicate that *Bmal1* silencing can potentiate PCB-induced alterations of endothelial permeability, autophagy, mitophagy, and metabolism. Overall, these results suggest that vascular toxicity of PCBs can be modulated by environmental factors that can influence the circadian rhythms.

**Grant Support:** Supported by the National Institutes of Health (NIH), grants MH128022, MH122235, MH072567, HL126559, DA044579, DA039576, DA040537, DA050528, and DA047157.

## A32 Investigation of blood–brain barrier changes in acute pancreatitis: a cell culture and clinical study

### Fruzsina R. Walter^1,2^, Andras Harazin^1^, Andrea E. Toth^1^, Szilvia Veszelka^1^, Ana R. Santa- Maria^1^, Judit P. Vigh^1^, Petra Pallagi^2^, Jozsef Maleth^2^, Laszlo Cervenak^3^, Agnes Kittel^4^, Peter Hegyi^2,5^, Zoltan Rakonczay^2^, Maria A. Deli^1^

#### ^1^Biological Research Centre, ELKH, Szeged, Hungary; ^2^Faculty of Medicine, University of Szeged, Szeged, Hungary; ^3^Department of Internal Medicine and Hematology, Research Laboratory, Semmelweis University, Budapest, Hungary; ^4^Institute of Experimental Medicine, ELKH, Budapest, Hungary; ^5^Institute of Translational Medicine, University of Pécs, Pécs, Hungary

##### Correspondence: Maria A. Deli - deli.maria@brc.hu

The central nervous system is affected in 10% of all severe inflammatory acute pancreatitis (AP) cases manifesting in pancreatic encephalopathy. Earlier research from our laboratory showed blood–brain barrier (BBB) permeability elevation in the rat non-invasive AP model induced by the administration of L-ornithine. Here we aim to explore these BBB integrity compromising mechanisms of L-ornithine using an in vitro rat primary cell-based BBB model. Our goal is also to test human serum samples from mild, moderate and severe cases of AP patients for BBB leakage markers and the effect of these sera on the barrier integrity of cultured human brain endothelial cells (hCMEC/D3 model).

Methods: (i) in vitro BBB modeling on Transwell inserts; (ii) functional tests – permeability, TEER, Ca2+ levels, mitochondrial activity, ROS/NO production, NFκB production; (iii) morphological tests: interendothelial junctions, mitochondria, ultrastructural changes with electron microscopy; (iv) surface charge measurements; (iv) ELISA assays for neuron specific enolase (NSE) and S100B presence in human sera. We found, that L-ornithine treatment decreased cell impedance and elevated permeability after 24 h treatment. Key interendothelial junctional and adhesion molecule expression and glycocalyx morphology was affected. ROS production was increased and mitochondrial network was also damaged while no intracellular Ca2+ or mitochondrial membrane potential alteration occurred. Elevated NSE and S100B levels were found in patients with mild, moderate and severe AP. Our results help to better understand the background of BBB alterations during AP and could lead to the development of future treatment strategies.

**Grant support:** Janos Bolyai Research Fellowship of the HAS, NKFIH OTKA PD-128480 and ITM UNKP-20–5-SZTE-672.

## A33 Disheveled interacts with claudin-5 and contributes to norrin-induced BRB restoration

### Monica Diaz Coranguez and David A. Antonetti

#### University of Michigan, Ophthalmology and Visual Sciences, Ann Arbor, MI, USA

##### Correspondence: David A. Antonetti—dantonet@med.umich.edu.

###### *Fluids and Barriers of the CNS* 2021, 18(1): A33

Previous studies reveal that norrin reverses VEGF-induced permeability in a β-catenin dependent pathway. Here, we explored the contribution of disheveled-1 (Dvl1) in norrin-induced blood-retinal barrier (BRB) restoration. We hypothesized that Dvl1 promotes tight junction (TJ) stabilization through both canonical and non-canonical signaling pathways. Analysis of BRB properties in primary bovine retinal endothelial cells (BREC) demonstrated that norrin was able to completely restore transendothelial electrical resistance (TEER) after VEGF. The knockdown of Dvl1 using siRNAs specifically reduced basal barrier properties and ablated norrin-induced barrier restoration, despite increased β-catenin signaling in knockdown samples. Similar results were found in flux assays of a 70 kDa RITC-dextran molecule, suggesting that Dvl1 is required for norrin-induced BRB restoration. Dvl immunofluorescence staining showed co-localization of Dvl1 with ZO-1 and claudin-5 at the TJ complex. Dvl1 and TJ proteins interaction analysis by co-immunoprecipitation of endogenous protein in BREC, demonstrated that Dvl1 interacts with both claudin-5 and ZO-1 and this interaction was most abundant in the presence of VEGF/norrin co-stimulation. Studies in HEK293 cells co-transfected with Dvl1 mutants and claudin-5 or ZO-1, reveal a requirement of the Dvl1 PDZ domain for this interaction. Transfection of the C-terminal fragment of Dvl1, containing the PDZ binding motif but not the DIX, PDZ or DEP domains, blocked Dvl1/claudin-5 interaction, increased basal permeability and prevented norrin-induced BRB restoration. Together, these results demonstrate that norrin signals though Dvl1 to stimulate barrier properties and suggest a non-canonical signaling role of Dvl1 in regulation of barrier properties through direct binding to claudin 5 and ZO-1.

**Grant support:** This work was supported by the NIH EY012021 (DAA), and Core grants, NIH P30EY007003 and DK020572, the Jules and Doris Stein Professorship from Research to Prevent Blindness (DAA), and the American Diabetes Association Minority Postdoctoral Fellowship Award #1–16-PMF-003 (MDC).

## A34 Exploring of the pro-angiogenic potential of pericytes in neurovascular repair upon stroke

### Maxime Bernard, Romain Menet, Sarah Lecordier, and Ayman ElAli

#### Department of Psychiatry and Neuroscience, Faculty of Medicine, Universite Laval; Research Center of CHU de Quebec-Universite Laval, Quebec City, QC, Canada Neuroscience Axis, CHU centre de recherche de Quebec (CHUL)

##### Correspondence: Ayman ElAli - Ayman.El-Ali@crchudequebec.ulaval.ca

Stroke is one of the leading causes of death and disability in the world. Unfortunately, still no efficient disease-modifying therapy exists to treat this neurological condition. Therapeutic angiogenesis using potent pro-angiogenic agents has already been shown to provide therapeutic benefits. However, the use of such agents is a double-edged strategy due to the increased risk of exacerbation of vascular leakage. Pericytes are specialized mural cells that play a key role in stabilizing the angiogenic vasculature and possess plastic properties, translated by cells’ capacities to change phenotype and function. Due to their angiogenic and plastic properties, pericytes constitute an attractive target for the development of novel interventions to promote neurovascular repair after stroke. Using novel molecular, cellular, and imaging approaches, our findings indicate that the recently identified platelet-derived growth factor (PDGF)-D plays an important role in promoting reactive pericyte function after stroke in mice. We first reported that PDGF-D expression is transiently and locally induced at the lesion site after stroke. Importantly, the intranasal delivery of recombinant PDGF-D in mice improved motor functions, which were accompanied with a decreased brain atrophy and reduced lesion size. Interestingly, these changes were associated with increased vascular density at the perilesional site as well as inside the lesion core in absence of hemorrhage. We found out that PDGF-D promoted the formation of new vessels that are adequately covered by pericytes. The overall results thus suggest that pericytes are an interesting target and a new tool for the development of new therapies against stroke.

## A35 Sirt1 contributes to preserve the blood–brain barrier integrity in CAA condition

### Ali F. Citalan-Madrid and Anuska V. Andjelkovic

#### Department of Pathology and Neurosurgery, University of Michigan Medical School, Ann Arbor, MI, USA

##### Correspondence: Ali F. Citalan-Madrid - citalanm@med.umich.edu.

###### *Fluids and Barriers of the CNS* 2021, 18(1): A35

CAA prevalence increases with age and is associate with AD. CAA is characterized by Aβ vascular deposition in cortex and leptomeninges, contributing to disruption of blood brain barrier (BBB). Sirt1 can attenuates the Aβ burden in Tg2576 transgenic AD mice model under caloric restriction. However, the role of Sirt1 in BBB dysfunction in the context of CAA is unknown. Here, we analyze the role of Sirt1 in BBB by using a CAA mouse model (TgSwDI) and generated TgSwDI mouse overexpressing Sirt1 (TgSwDI-Sirt1over). Amyloid accumulation was presented in > 80% of brain blood vessels at 6 and 12 months in TgSwDI. Additionally, T2* MRI evaluation and Prussian blue staining showed multiple hemorrhagic lesions in TgSwDI mice from 6 months. WB analysis showed reduced expression of claudin-5 and ZO-1 in brain blood vessels alongside with increased BBB permeability for inulin (5 kDa). IF-staining of 250-μm thick brain tissue sections showed a reduction of fluorescence intensity, defragmented pattern, and loss of colocalization for claudin-5 and ZO-1 of brain capillaries in TgSwDI mice. Interestingly, preserved expression of Sirt1 in TgSwDI-Sirt1over mice showed a reduction in Aβ vascular deposition and highly preservation of ZO-1 and claudin-5 in brain blood vessels in comparison with TgSwDI mice. Thus, Sirt1 can contributes to regulate and preserve the BBB integrity in CAA condition.

**Grant Support:** This work was supported by NIH grant number NIH RF1 AG064957 (AVA); AFCM received a scholarship from Secretariat of Education, Science, and Technology Innovation (SECTEI) of Mexico City.

## A36 Protocadherin gamma C3 (PCDHGC3) and its role at the blood–brain barrier

### Victoria Kaupp, Christina Dilling, Patrick Meybohm, Malgorzata Burek

#### University of Würzburg, University Hospital Würzburg, Department of Anaesthesiology, Intensive Care, Emergency and Pain Medicine, Würzburg, Germany

##### Correspondence: Victoria Kaupp - Kaupp_V@ukw.de

###### *Fluids and Barriers of the CNS* 2021, 18(1): A36

Brain microvascular endothelial cells play a critical role in the maintenance and tightness of the blood–brain barrier through the formation of tight junctions, which leads to a potent restriction of paracellular diffusion. Occludin, a transmembrane protein with two extracellular loops and N- and C-termini in the cytoplasm, is an integral part of tight junctions, the role of which, however, has not yet been sufficiently investigated. To elucidate its contribution to the blood–brain barrier, we looked for new interaction partners of occludin, which led us to protocadherin gamma C3 (PCDHGC3), among others. PCDHGC3 is highly expressed in the central nervous system including brain microvascular endothelial cells, but has not yet been characterised for interaction with tight junction proteins at the blood–brain barrier. In this study, we analysed the interaction of PCDHGC3 with occludin using transformation and transfection methods. The cDNA of PCDHGC3 and occludin were amplified in DH5α, co-transfected into mammalian cells and immunoprecipitated with anti-occludin antibody. Western Blot analysis showed a specific interaction between PCDHGC3 and occludin, using non-immunoprecipitated cell lysate as an endogenous control. These methods are currently used to analyse mutants of occludin whose N-terminus is necessary for the interaction, as well as several other interaction partners. Since PCDHGC3 is mostly expressed in the brain, such an interaction with occludin could be specific for the blood–brain barrier endothelium and could contribute to the specialized character of brain endothelial cells. Our results provide a basis for further investigations regarding the role of occludin and PCDHGC3 at the blood–brain barrier.

## A37 Claudin-1 overexpression remodels tight junction complex, leading to blood–brain barrier dysfunction in aging

### Gabriela Martinez-Revollar^1^, Sarah Jacobs^2^, Ali F Citalan-Madrid^1^ and Anuska V. Andjelkovic^1,3^

#### ^1^Department of Pathology, Molecular, ^2^Cellular & Development Biology Program ^3^Department of Neurosurgery, University of Michigan Medical School, Ann Arbor, MI, USA

##### Correspondence: Anuska V Andjelkovic- anuskaa@med.umich.edu

###### *Fluids and Barriers of the CNS* 2021, 18(1): A37

Aging is associated with a decline in blood brain barrier (BBB) integrity, leading to cognitive dysfunction and neurodegeneration due to capillary leakiness. Claudin-1 is one of the proteins whose expression increases in leaky BBB. Here we investigated whether claudin-1 expression in mice brain microvessels affects BBB integrity in aging. The claudin-1 expression was analyzed in vivo in young (4–6 months) and aging (18–20 months) mice and in vitro using a senescence model (continuous passage) of mice primary brain microvascular cells (mBEMC). The senescence phenotype was confirmed using senescence markers such as β-galactosidase activity, phosphorylated H2A.X and DAPI staining associated to DNA damage. BBB integrity, both in vitro (evaluated by impedance-based cell monitoring) and *in viv*o (clearance of inulin 5 kDa tracer) was compromised in the aging group. We observed a markedly higher expression of claudin-1 and a decrease of claudin-5 expression in mBEMC both in vitro and in vivo*.* Morphologically, aging mBEMC showed discontinued ZO-1 membrane staining with “ruffles” appearance and novel claudin-1 strands, indicating that claudin-1 contributes to strand formation in senescence cells. Relatedly, ZO-1/claudin-5 interaction was lost, instead claudin-1/ZO-1 and claudin-1/claudin-5 interaction increased in “old” mBEMC. The role of claudin-1 in aging process was further confirmed by depletion of claudin-1 in aging mBEMC. This rescued the phenotype of the mBEMC, improving the barrier integrity and restoring claudin-5/ZO-1 interaction, in addition senescence markers were decreased. Our findings provide, for the first time, a possible role of claudin-1 in the functional impairment of BBB in elderly mouse brain.

## A38 Defining brain pericytes as a novel HIV reservoir

### Oandy Naranjo, Silvia Torices, Luc Bertrand, and Michal Toborek

#### Department of Biochemistry and Molecular Biology, 1011 NW 15th Street; Gautier Building, Room 528; University of Miami Miller School of Medicine, Miami, FL 33,136–1019, USA

##### Correspondence: Michal Toborek - mtoborek@med.miami.edu

HIV-1-infected individuals are at a higher risk for non-AIDS related co-morbidities, including cerebrovascular and neurological diseases. These pathologies may be driven, at least in part, by low levels of viral replication that persist in HIV-infected brains, which can lead to immune activation, chronic inflammation, and viral reactivation. Experiments on microglia, astrocytes, and brain pericytes indicate that these cells are all capable, to different degrees, to harbor HIV infection. We have pioneered research on HIV-1 infection in brain pericytes, and indicated that these cells possess the receptor profile enabling HIV-1 infection. Recent evidence on pericyte ontogeny identified that a substantial subpopulation of brain pericytes originates from myeloid progenitors. We hypothesize that brain pericytes are a key, albeit previously unrecognized, cell type for the formation of HIV-1 reservoirs in the CNS. Indeed, our research indicates that BBB pericytes can be a target of HIV-1 infection able to support productive HIV-1 replication. Interestingly, HIV-1 infection of pericytes can be regulated by occludin and a number of occludin regulated host genes. In addition, we have evidence that BBB pericytes are prone to establishing a latent infection, which can be reactivated by a mixture of histone deacetylase inhibitors in combination with TNF. HIV-1 infection of BBB pericytes has been confirmed in human postmortem samples of HIV-1-infected brains. Overall, our study indicates that BBB pericytes can be a previously unrecognized HIV-1 target and reservoir in the brain.

**Grant Support:** Supported by the NIH grant MH128022.

## A39 A 20 kDa isoform of Connexin-43 (Cx43-20 kDa) mediates transcriptomic and epigenetic changes in brain endothelial cells

### Chelsea M. Phillips^1^, Katherine M. Bonefas^1^, Shigeki Iwase^2^, Anuska V. Andjelkovic^3^

#### ^1^Neuroscience Graduate Program, ^2^Department of Human Genetics, ^3^Department of Pathology and Neurosurgery, University of Michigan, Ann Arbor, MI, USA

##### Correspondence: Chelsea Phillips - chelsphi@umich.edu

###### *Fluids and Barriers of the CNS* 2021, 18(1): A39

Six N-terminally truncated isoforms of Connexin-43 (Cx43), a gap junction protein, have recently been identified. Of these isoforms, a 20 kDa isoform (Cx43-20 kDa) has the highest expression and is involved in cellular processes such as Cx43 trafficking, cytoskeletal organization, mitochondrial transport, and transcriptional regulation during development. We have previously demonstrated that Cx43-20 kDa alters the phenotype of mouse brain endothelial cells (mBECs), as Cx43-20 kDa overexpression 1) increases Cx43 expression, 2) remodels tight junction (TJ) and gap junction (GJ) complexes, and 3) increases GJ intercellular communication. Our study objective is to determine the mechanism through which Cx43-20 kDa alters the phenotype of brain endothelial cells. To determine how Cx43-20 kDa alters the phenotype of brain endothelial cells, mRNA-sequencing (RNA-seq) and reduced representation bisulfite sequencing (RRBS) were conducted on control mBECs and Cx43-20 kDa-overexpressing mBECs to determine transcriptome and methylome changes, respectively. Protein expression of differentially expressed genes (DEGs) of interest was determined through Western blot. Cx43-20 kDa overexpression alters the endothelial cell transcriptome, with gene ontology analysis revealing that DEGs are involved in immune processes, cell–cell junctions, and the extracellular matrix. RNA-seq analysis revealed Cx43-20 kDa overexpression decreases transcript expression of DNA methyltransferase 3A (DNMT3A), a de novo DNA methyltransferase, which was confirmed through Western blot. RRBS analysis revealed that Cx43-20 kDa overexpression alters the methylome, with differently methylated cytosines (DMCs) located on genes encoding junction proteins and anchoring junctions. Collectively, our data demonstrates Cx43-20 kDa mediates changes in the transcriptome and methylome of mBECs.

**Grant Support:** This work was supported by NIH R21NS098066 and R21NS11120501; CMP was supported by NIH T-32-NS076401 and Rackham Regents Fellowship.

## A40 Chronic exposure to prescription opioids interrupts the integrity of blood–brain barrier and increases the severity of ischemic stroke

### Enze Sun, Cherisse Asher Dawn Charles and Michal Toborek

#### Department of Molecular Biology and Biochemistry, Miller School of Medicine, University of Miami, Miami, USA

##### Correspondence: Michal Toborek—mtoborek@med.miami.edu

###### *Fluids and Barriers of the CNS* 2021, 18(1): A40

The epidemic of opioid abuse endangers not only the public health but also the social and economic welfare. Growing evidences indicate that abuse of opioids, including prescription pain relievers, may contribute to the development of stroke and/or affect stroke recovery. Given the pivotal role of blood–brain barrier (BBB) in the context of stroke, we hypothesize that the chronic exposure to prescription opioids could compromise the integrity of the blood–brain barrier (BBB). In the mouse model exposed to two common prescription opioids, morphine and oxycodone, with escalating dosages, we detected increased BBB permeability, fragmentation of tight junction distribution and downregulated expression of occludin and ZO-1, two major tight junction proteins. Middle cerebral artery occlusion (MCAO) model also proved that the exposure of opioids would exacerbate the severity of ischemic stroke. Further evidence showed that the elevated levels of pro-inflammatory cytokines like TNF-α and oxidative stress induced by the opioid exposure in the in vitro model contributed to the mitochondrial dysfunction, which might play a key role in the alteration of BBB in vivo. These results provide new insights to the long-term side effect of prescription opioids on the stability of neurovascular unit.

**Grant Support:** Supported by the National Institutes of Health (NIH), grants DA044579, DA039576, DA040537, DA050528, DA047157, MH128022, MH122235, MH072567, and HL126559, DA044579, DA039576, DA040537, DA050528, and DA047157.

## A41 Tackling stroke… in vitro: characterization of a triculture human Blood–Brain Barrier model and its suitability for nanogel studies

### Eleonora Rizzi^1^, Federico Traldi^2^, Susana Simoes^3^, Susana Rosa^3^, Francesca Tomatis^3^, Marie-Pierre Dehouck^1^, Fabien Gosselet^1^, Lino Ferreira^3^, Marina Resmini^2^, Caroline Mysiorek^1^

#### ^1^Artois University, The Blood–Brain Barrier Laboratory (LBHE) UR 2465, Lens France; ^2^Queen Mary University of London, School of Biological and Chemical Sciences, London, United Kingdom; ^3^Universtiy of Coimbra, CNC- Center for Neuroscience and Cell Biology, CIBB – Center for Innovative Biomedicine and Biotechnology, Coimbra, Portugal

##### Correspondence: Eleonora Rizzi - eleonora.rizzi@univ-artois.fr

###### *Fluids and Barriers of the CNS* 2021, 18(1): A41

As the second leading cause of death and long-term disabilities worldwide [1], stroke needs to be further studied to increase the range of treatment available to decrease its pathological outcome. Caused by the formation of an obstruction in a brain vessel, the ischemic stroke is linked to shortage of nutrient and oxygen rich blood flow in the downstream region causing an increase in Blood–Brain barrier (BBB) permeability and parenchymal damage. Being a multifactorial disease, it is possible to use different strategies to tackle the different pathological phases [2]. Among the different novel strategies, the use of nanoparticles (*i.e.* nanogels [3]) is getting more and more interest [4]. In vitro studies and BBB models using human cells that mimic the in vivo environment during a stroke accident are essential to be able to understand cells mechanism and translate results in clinic. The BBB is formed by specialized Endothelial Cells (ECs) in the brain microvasculature and are surrounded by other cell types as Brain Pericytes and Astrocytes to create a specific microenvironment within which cells communication and interaction are essential for the maintenance of a physiological environment but also for the occurrence of pathologies. In this study we developed and characterized a triculture human BBB in vitro model submitted to Oxygen and Glucose Deprivation (OGD) [5] for 6 h and Reoxygenation (R) for 24 h. This BBB model has been characterized for barrier tightness, intracellular energy stores and gene expression as well as for nanogels toxicity, crossing and internalization, giving promising tools for further studies.

**Grant support:** This project has received funding from the European Union’s Horizon 2020 research and innovation programme under grant agreement No 764958.

**References**

1. Donkor ES. Stroke in the 21(st) Century: A Snapshot of the Burden, Epidemiology, and Quality of Life. Stroke Res Treat. 2018;2018:3238165. https://doi.org/10.1155/2018/3238165.
2. Bernardo-Castro S, Albino I, Barrera-Sandoval AM, Tomatis F, Sousa JA, Martins E et al. Therapeutic Nanoparticles for the Different Phases of Ischemic Stroke. Life (Basel). 2021;11(6). https://doi.org/10.3390/life11060482.
3. Salinas, Y., Castilla, A. M. & Resmini, M. An l-proline based thermoresponsive and pH-switchable nanogel as a drug delivery vehicle. Polym. Chem. 2018;9, 2271–2280.
4. Gosselet F, Loiola RA, Roig A, Rosell A, Culot M. Central nervous system delivery of molecules across the blood–brain barrier. Neurochem Int. 2021;144:104952. https://doi.org/10.1016/j.neuint.2020.104952.
5. Mysiorek C, Culot M, Dehouck L, Derudas B, Staels B, Bordet R et al. Peroxisome-proliferator-activated receptor-alpha activation protects brain capillary endothelial cells from oxygen–glucose deprivation-induced hyperpermeability in the blood–brain barrier. Curr Neurovasc Res. 2009;6(3):181–93. https://doi.org/10.2174/156720209788970081.

## A42 The cell-penetrating peptide Tat facilitates efficient endocytic uptake of a therapeutic peptide into brain endothelial-like monolayers but limited barrier permeation

### Emma Lisa Al Humaidan^*^, Sidse Lund Pedersen^*^, Burak Ozgür, Birger Brodin, Mie Kristensen

#### Faculty of Health and Medical Sciences, Department of Pharmacy, University of Copenhagen, Denmark

*Authors contributed equally.

##### Correspondence: Mie Kristensen - mie.kristensen@sund.ku.dk.

###### *Fluids and Barriers of the CNS* 2021, 18(1): A1

**Objectives:** Cell-penetrating peptides may serve as shuttles for delivery of brain therapeutics across the blood–brain barrier (BBB). An example is the peptide NR2B9c1, which has been conjugated to the cell-penetrating peptide Tat2 to facilitate BBB permeation and internalization into neurons. NR2B9c may relieve neuronal damage after stroke upon target engagement in neurons. The aim of this study was to investigate BBB permeation of Tat-NR2B9c, and whether that happens via paracellular- or transcellular transport; or a combination.

**Methods:** A human stem cell-based BBB model (BIONi-010C3) cultured on transwell inserts was used for the experiments. Barrier integrity was monitored with a CellZscope. Transport studies were performed with flourescently labelled Tat-NR2B9c and radiolabelled mannitol. Tight junction protein expression was studied using immunocytochemistry and western blotting. The mechanism of cellular Tat-NR2B9c uptake was performed using live cell microscopy.

**Results:** Tat-NR2B9c efficiently adhered to the cellular plasma membrane and internalized via endocytosis, whereas limited amounts of Tat-NR2B9c permeated the BIONi-010C monolayers. Tat-NR2B9c affected the barrier properties, but without giving rise to increased mannitol permeation or changes in tight junction protein expression.

**Conclusions:** Tat facilitates cellular internalization of the therapeutic moiety NR2B9c, whereas its ability to permeate the BBB upon NR2B9c conjugation is limited. The mechanism of BBB permeation is likely a combination of paracellular transport and transcytosis.

**References**

1. Aarts M, Liu Y, Liu L, Besshoh S, Arundine M, Gurd JW et al. Treatment of ischemic brain damage by perturbing NMDA receptor- PSD-95 protein interactions. Science. 2002;298(5594):846-50. https://doi.org/10.1126/science.1072873.
2. Vives E, Brodin P, Lebleu B. A truncated HIV-1 Tat protein basic domain rapidly translocates through the plasma membrane and accumulates in the cell nucleus. J Biol Chem. 1997;272(25):16010-7. https://doi.org/10.1074/jbc.272.25.16010.
3. Goldeman C, Andersen M, Al-Robai A, Buchholtz T, Svane N, Ozgur B et al. Human induced pluripotent stem cells (BIONi010-C) generate tight cell monolayers with blood-brain barrier traits and functional expression of large neutral amino acid transporter 1 (SLC7A5). Eur J Pharm Sci. 2021;156:105577. https://doi.org/10.1016/j.ejps.2020.105577.

## A43 Primary brain capillary endothelial cell monolayers cultured under hypoxic conditions maintain barrier integrity and display increased expression of Glut1 (SLC2A1), Lat1(SLC7A5) and transferrin receptor 1(TFRC)

### Burak Ozgur, Hans Christian Cederberg Helms, Erica Tornabene and Birger Brodin

#### Department of Pharmacy, The Faculty of Health and Medical Sciences, University of Copenhagen, Universitetsparken 2, DK-2100, Copenhagen, Denmark

##### Correspondence: burak.ozgur@sund.ku.dk

**Background and aim: **Brain capillary endothelial cells (BCECs) are exposed to a hypoxic environment during early brain development. However, little is known of the effect of hypoxia on barrier properties of the endothelium and blood-brain barrier (BBB) function. A number of studies have suggested breakdown of the barrier function, whereas others have claimed beneficial effects of the hypoxic conditions. The study aimed at investigating if primary BCECs cultured under hypoxic conditions maintained BBB function.

**Method: **Primary monocultures of bovine BCECs were cultured on permeable supports under normoxic (90% atmospheric air-10% CO2) and hypoxic (1% O2, 10% CO2 and 89% N2) conditions for 6 days. The tightness of endothelial cell monolayers was investigated using electrical resistance measurements and expression of BBB phenotype markers was investigated using RT-PCR, western blotting and immunohistochemistry.

**Results: **In cell monolayers exposed to hypoxia, we observed translocation of HIF-1 alpha from cytosol to nucleus, indicating a cellular hypoxic response via the HIF-signaling pathways. Hypoxia throughout a 6-day culture period did not affect monolayer integrity, as judged from electrical resistance measurements. Localization of claudin-5 and ZO-1 was not affected by hypoxia. GLUT-1, LAT-1 and TfR were significantly upregulated on the mRNA level in cells cultured under hypoxic conditions. The upregulation of TfR and GLUT-1 could be confirmed at protein level. Cells cultured under hypoxia displayed a higher glucose uptake, as compared to normoxic cultures. The effects of hypoxia on the endothelial cells could be mimicked by stimulating cells with 10 μM of the HIF-1α stabilizing compound deferoxamine (DFO), under normoxic conditions, indicating that the effect were mediated via HIF-1α.

**Conclusion: **In conclusion, BCECs develop monolayers with well-developed tight junctional complexes under hypoxic conditions, and increase expression of GLUT-1, LAT-1 and TfR. This supports the notion that the BBB function of the brain capillaries is present at the early stages of brain vasculature development, where the brain environment is hypoxic.

## A44 Characterization of human brain pericyte-derived small extracellular vesicles

### Camille Menaceur^1^, Paulo Marcelo^2^, Andreas Karner^3,4^, Guillaume Van Niel^5^, Bastien Leger^6^, Yasuteru Sano^7^, Fumitaka Shimizu^7^, Takashi Kanda^7^, Jaroslav Jacak^3,4^, Fabien Gosselet^1^, Laurence Fenart^1^ and Julien Saint-Pol^1^

#### ^1^Laboratoire de la Barrière Hémato‐Encéphalique (LBHE), Univeristy Artois, UR 2465, 62300 Lens, France; ^2^Plateforme d'Ingénierie Cellulaire et d'Analyses des Protéines, Centre Universitaire de Recherche en Santé, Amiens, France; ^3^ Institute of Applied Physics, Johannes Kepler University Linz, 4040 Linz, Austria; ^4^ Upper Austrian University of Applied Sciences, Campus Linz, 4020 Linz, Austria; ^5^Université de Paris, Institute of Psychiatry and Neuroscience of Paris (IPNP), INSERM U1266, “Endosomal dynamic in neuropathies”, Paris, France; ^6^ University Artois, CNRS, Centrale Lille, ENSCL, UNiv. Lille, UMR 8181, Unité de Catalyse et de Chimie du Solide (UCCS), F-62300 Lens, France; ^7^ Department of Neurology and Clinical Neuroscience, Yamaguchi University Graduate School of Medicine, Ube, Japan

##### Correspondence: Julien Saint-Pol - julien.saintpol@univ‐artois.fr

###### *Fluids and Barriers of the CNS* 2021, 18(1): A44

The brain compartment is protected by the blood-brain barrier (BBB) ​​which limits the passage of circulating molecules into the central nervous system (CNS), thus preserving cerebral homeostasis. The BBB phenotype is carried by the endothelial cells (ECs) forming the wall of the cerebral microvessels and resting on a basal lamina common with brain pericytes (PCs), the whole being surrounded by a continuous sleeve of astrocyte end-feet. This structure is also in communication with neurons and other types of glial cells, which altogether are referred to as the neurovascular unit (NVU). During embryogenesis, the establishment of the BBB phenotype is possible through cell-cell communication between the components of the UNV. PCs have a major role in the induction of the phenotype from the early stages of its establishment. Indeed, they (i) induce the specific localization of the proteins of the basement membrane of the ECs, (ii) participate in the structuring of the junctions between the ECs and in the restriction of vesicular transport, and (iii) are also essential for the maintenance of the BBB phenotype. Communication between these different cell types is achieved by the exchange of soluble factors, and potentially by extracellular vesicles (EVs), of which exosomes (nanoscopic vesicles 30 to 100 nm in diameter of endosomal origin) are part. Moreover, exosomes have been described as communication vectors capable of regulating the BBB junctional complexes by through miRNA from neurons to ECs. This study therefore aims to characterize *in vitro* the PCs-derived EVs and particularly the small EVs. EVs features are checked by dynamic light scattering (DLS), zeta potential measurement, atomic force microscopy (AFM) and transmission electron microscopy (TEM), and the protein composition is evaluated by mass spectrometry. This mapping is essential for further studies focused on the role of these EVs in establishing and maintaining the BHE main features.

## A45 The protein interaction between breast cancer cells and brain microvascular endothelial cells can promote the development of metastases

### Ramon Handeron Gomes Teles¹, Ana Sayuri Yamagata¹, Nicolas Jones Villarinho¹, Patrick Meybohm², Malgorzata Burek², Vanessa Morais Freitas¹

#### ¹Department of Cell and Developmental Biology, Institute of Biomedical Sciences (ICB), University of São Paulo, São Paulo, Brazil; ²Department of Anaesthesiology, Intensive Care, Emergency and Pain Medicine, University Hospital Würzburg, Würzburg, Germany

##### Correspondence: Ramon H. Gomes Teles - ramonteles@usp.br

###### *Fluids and Barriers of the CNS* 2021, 18(1): 45

Breast cancer is the leading cause of cancer-related death in women worldwide, with around 90% due to the development of metastases. This is a significant concern in oncology because it is difficult to recognize and treat the metastases. Breast cancer metastases present tropism to some organs, one of which is the brain, constituting the main challenge to be treated. Thus, it is important to understand the biological mechanisms that promote metastasis in the brain. In this way, we wanted to investigate the association between proteins from circulating breast cancer cells and proteins in the brain endothelial cell surface, as this is one of the most important steps in the metastatic cascade. To achieve this goal, we analyzed proteomics datasets of MDA-MB-231 and HBMEC cells and selected the proteins that were differently expressed compared to their controls (MCF-10A and HUVEC), followed by testing the interaction between them. First, we found a total of 397 proteins that were filtered and integrated into a cluster of 45, which showed a significant correlation with the molecular function such as catalytic activity and protein binding associated with some important biological process during metastatic cell invasion, e.g. cell adhesion and locomotion. Most of the protein classes considered metabolite interconversion enzymes such as dehydrogenases and glycosyltransferase. These partial results suggest that proteins expressed differently in breast cancer cells have a strong correlation with proteins that are uniquely found in the brain vascular bed which are crucial for the metastatic invasion.

## A46 Proteomic landscape of brain endothelial extracellular vesicles: remodeling by HIV-1 and amyloid beta

### Ibolya E. Andras, Brice B. Sewell and Michal Toborek

#### Department of Biochemistry and Molecular Biology, University of Miami School of Medicine, Miami, FL 33136-1019, USA

##### Correspondence: Ibolya Andras - IAndras@med.miami.edu

###### *Fluids and Barriers of the CNS* 2021, 18(1): A46

HIV-infected brains have an increased amyloid beta (Aβ) deposition. The blood-brain barrier (BBB) may be critical for the brain’s Aβ homeostasis and could contribute to Aβ accumulation. Extracellular vesicles (EVs) are important players in Aβ pathology. Interestingly, HIV-1 increased EV release from brain endothelial cells and altered their EV-Aβ levels. The mechanisms of this EV-mediated Aβ pathology are not clear. EVs carrying various cargo molecules, including a complex set of proteins, can significantly influence the biology of surrounding neurovascular unit cells. In this work, we examined how exposure to HIV, alone or together with Aβ, affects the surface and total proteomic landscape of brain endothelial EVs. This unbiased approach gave us an unprecedented, high-resolution insight into these changes. Our results show that HIV and Aβ profoundly remodel the complex proteome of brain endothelial EVs, altering the pathway networks and functional interactions among proteins. These mechanisms may contribute to the EV-mediated amyloid pathology in the HIV-infected brain.

**Grant support:** This work was supported by the Florida Department of Health grant 8AZ24 and the National Institutes of Health (NIH), grants MH072567, MH098891, HL126559, DA039576, DA040537, DA044579, and DA047157. We acknowledge support from the Miami Center for AIDS Research (CFAR) at the University of Miami Miller School of Medicine, which is funded by a grant (P30AI073961) from the National Institutes of Health (NIH).

